# Unraveling a novel therapeutic facet of Etravirine to confront Hepatocellular Carcinoma via disruption of cell cycle

**DOI:** 10.1038/s41598-025-87676-3

**Published:** 2025-02-10

**Authors:** Gouri Nair, G. R. Saraswathy, Jesil Mathew Aranjani, Rafwana Ibrahim

**Affiliations:** 1 Department of Pharmacology, Faculty of Pharmacy, M. S. Ramaiah University of Applied Sciences, Bangalore, Karnataka India; 2 Department of Pharmacy Practice, M. S. Ramaiah University of Applied Sciences, Bangalore, Karnataka India; 3 Department of Pharmaceutical Biotechnology, Manipal College of Pharmaceutical Sciences, Manipal Academy of Higher Education, Manipal, Karnataka India

**Keywords:** Hepatocellular Carcinoma, Drug repurposing, CCNA2, CDK1, CDK2, Etravirine, Virtual drug screening, Computational biology and bioinformatics, Cancer, Liver cancer

## Abstract

Hepatocellular Carcinoma (HCC) is a malignancy with high mortality rates and limited treatment options. This study aimed to unearth the repurposable potential of FDA-approved drugs against specific genetic targets governing the HCC pathological pathways. The transcriptomics microarray datasets were explored to retrieve the HCC specific differentially expressed genes, and the significant genes were fed in Search Tool for the Retrieval of Interacting Genes/Proteins (STRING) database to capture the protein-protein interactions, which were visualized in Cytoscape. This revealed CCNA2, a cell cycle regulator, as a potential target, which mediates its action by interacting with CDK1 and CDK2. Further, with the intention of identifying inhibitors for CDK1 and CDK2, a drug library was created, and the drugs were virtually screened against their respective targets via molecular docking and dynamics studies. This captured the binding affinity of Steviolbioside towards CDK1 and Etravirine and Fludarabine towards CDK2. In vitro, validation confirmed the cytotoxic potential of Etravirine and Fludarabine in Huh-7 cell lines. Further, enzymatic assays, gene expression analysis, and cell cycle analysis signified the anti-proliferative potential of Etravirine in Huh-7 cells via inhibition of CDK2. In this drug repurposing venture, Etravirine, a non-nucleoside reverse transcriptase inhibitor indicated for the treatment of HIV, emerged as a promising candidate for HCC treatment. The findings warrant further preclinical and clinical investigations to ascertain the repurposable potential of Etravirine against HCC, particularly in patients with viral infections.

## Introduction

Drug repurposing has gained significant traction in recent years as this stratagem systematically orchestrates and leverages cutting-edge technologies to disinter novel therapeutic targets, pathways and indications of existing drugs and abandoned drugs with utmost precision. For instance, Thalidomide, a sedative drug that was outlawed in several nations owing to its catastrophic impact on foetal development, which prompted the establishment of a global pharmacovigilance program, is now studied for its novel therapeutic indications. Currently, this drug is approved by the US FDA for the management of leprosy and multiple myeloma with a special labeling of contraindication in pregnancy^1^.

Drug repurposing is of paramount importance in oncotherapeutics due to intolerable adverse effects and emerging therapeutic resistance, especially in cancers with limited therapeutic options and poor prognosis. Hepatocellular Carcinoma (HCC) is one such aggressive malignancy of global concern with a high mortality rate. In 2020, liver cancer ranked as the sixth most regularly detected cancer and the third main cause of most cancer-related fatalities globally. This devastating disease accounted for over 9,00,000 new diagnoses and more than 8,30,000 deaths^2^. There are reports stating about 9.3 new instances of liver cancer worldwide for every 100,000 population, with an 8.5% death rate^3^. Therapeutic options for HCC include immune checkpoint inhibitors (Nivolumab and Pembrolizumab), multikinase inhibitors (Regorafenib, Lenvatinib, Cabozantinib, and Ramucirumab), and a combination of Bevacizumab/Atezolizumab and Tremelimumab/Durvaluma; however, economic constraints add limitations. Despite numerous approved treatment modalities, the development of effective therapeutic strategies is still an urgent requirement owing to the heterogeneous genetic and epigenetic alterations contributing to development, invasiveness and progression of the disease^4^.

This study is a drug repurposing venture that is focused on disinterring the repurposable potential of FDA-approved drugs against specific genetic targets governing the HCC pathological pathways. At the outset, transcriptomics big data was explored, leveraging bioinformatic algorithms to identify differential gene expression in HCC. Based on statistical significance, robust genes were mapped with proteins that are considered as potential drug targets. Subsequently, standard inhibitors or literature-derived inhibitors specific to the shortlisted disease targets were collated to create a comprehensive drug library by the inclusion of other FDA drugs sharing structural and side effect similarities. Further, computational approaches were employed to establish drug-target interactions virtually. Drugs that displayed significant interactions and stability within the binding pockets of the target proteins were validated for their cytotoxic and cell cycle-arresting properties in Huh-7 cell lines.

## Result

### Phase 1: identification of potential targets for HCC and pathway analysis

Datasets comprising developmental stages of HCC (normal, cirrhosis, and HCC) were chosen to be the focus of the investigation. The potential druggable targets for HCC and the carcinogenic cascade from preneoplastic lesion to neoplasm were identified by analyzing the gene expression profile.

### Omics-based differential gene expression analysis

**Fig. 1:**
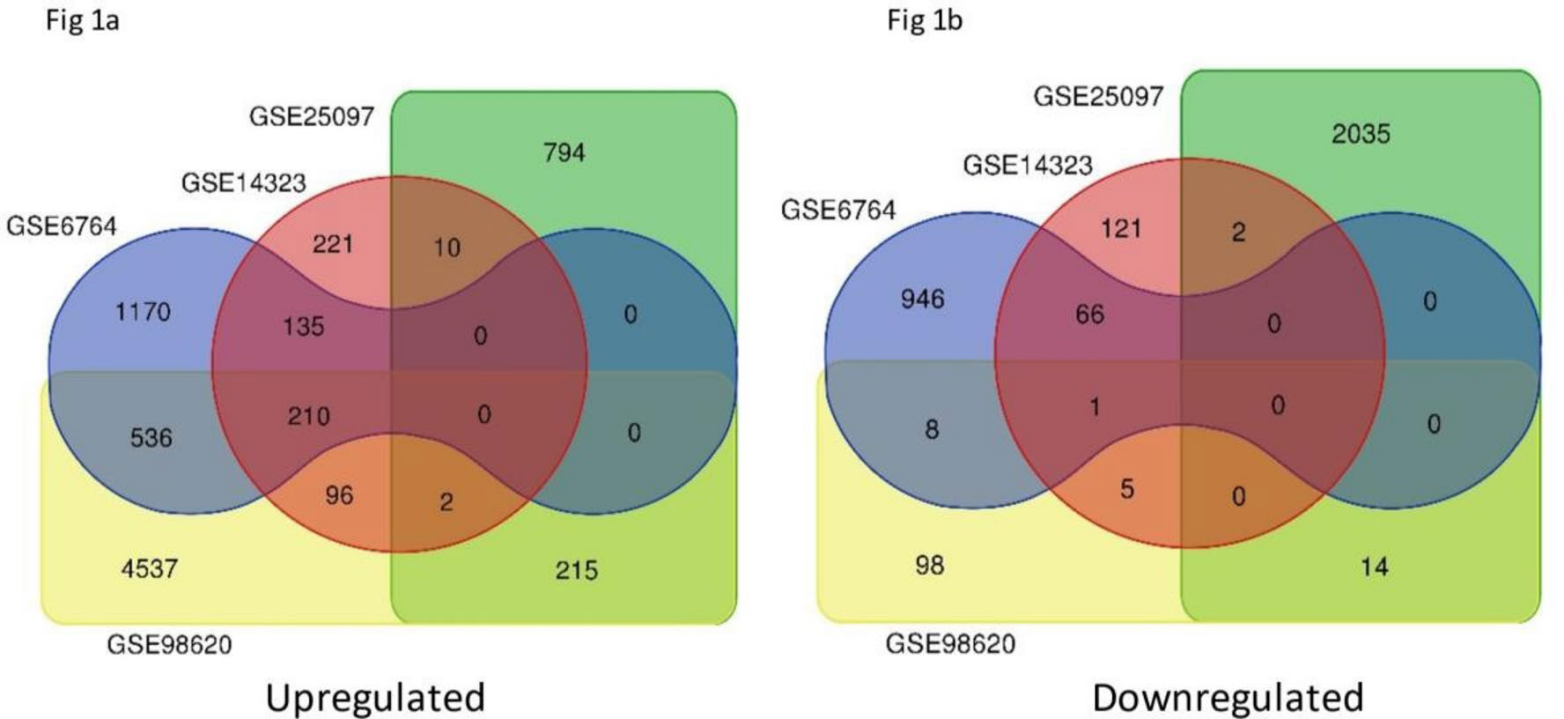
Venn diagram of common upregulated and downregulated DEGs in normal Vs cirrhosis based on the criteria “P-value < 0.05 with Log FC > 1”.

**Fig. 2:**
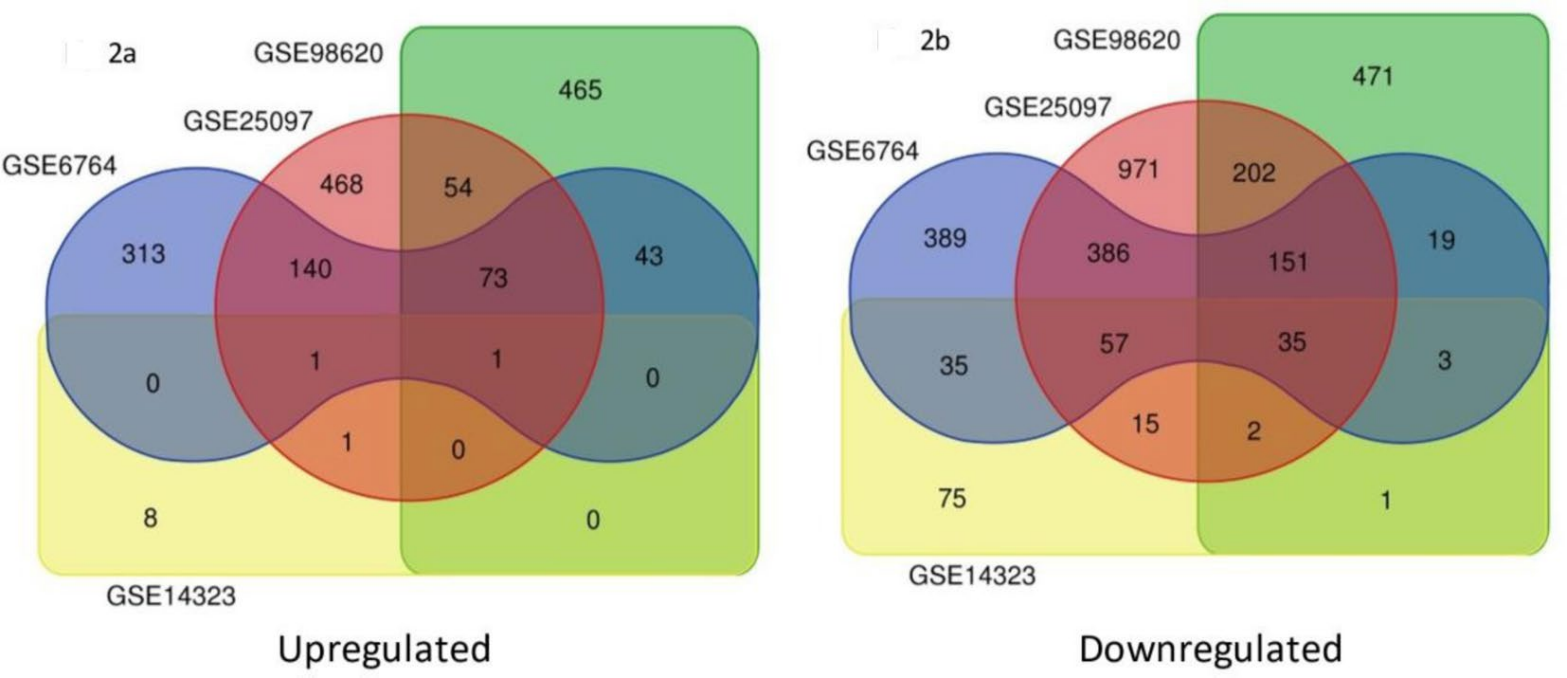
Venn diagram of common upregulated and downregulated DEGs in cirrhosis Vs HCC based on the criteria “P-value < 0.05 with Log FC > 1”.

In normal vs. cirrhosis, 210 common upregulated DEGs, and one common downregulated DEG (Fig. 1). In cirrhosis vs. HCC, total of (73 + 1 + 1) 75 common upregulated DEGs were chosen. Similarly, total of (35 + 151 + 57 + 3 + 2) 248 common downregulated DEGs were shortlisted (Fig. 2).

### Protein-protein interactions

**Fig. 3:**
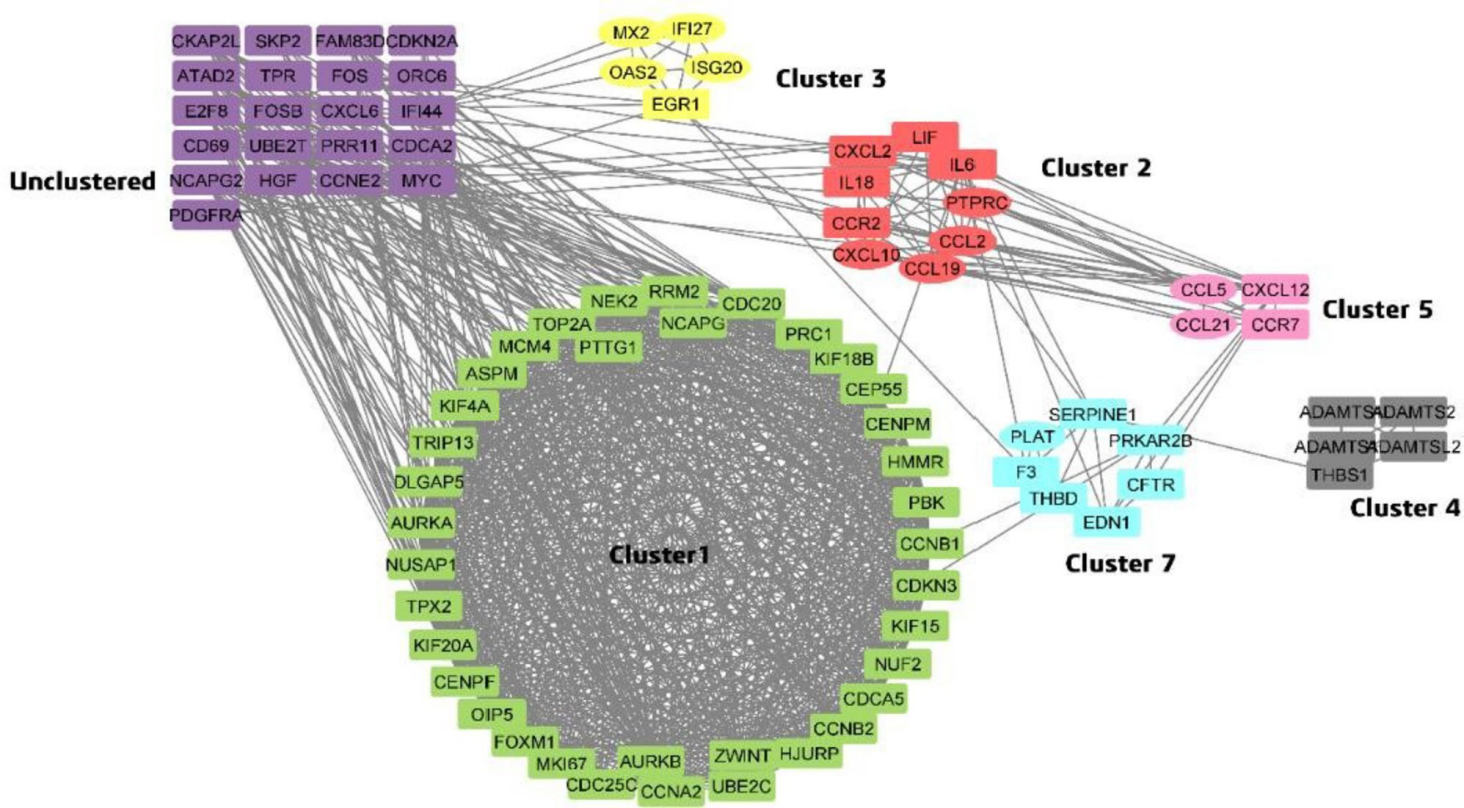
PPI of DEGs in different clusters. Elliptical shaped nodes indicate proteins of Normal Vs Cirrhosis and rectangle-shaped nodes indicate proteins of HCC Vs cirrhosis.

Shortlisted common DEGs from Normal Vs Cirrhosis and Cirrhosis Vs HCC were assessed via the STRING tool to identify the physical and functional associations among the proteins of DEGs. The protein interactions were further visualized through Cytoscape, which revealed 2820 edges and 213 nodes. Cyclin A2 (CCNA2) exhibited the highest node degree of 49, with an MCODE score of 28 in cluster 1, and hence, it was identified as a potential DEG (Fig. 3) (Table 1).

**Table 1:** Cluster analysis of DEGs.

Node degree	MCODE Cluster	MCODE Score	Gene symbol	Group
49	Cluster 1	28.6704546	CCNA2	C vs. HCC
46	Cluster 1	28.6704546	CCNB1	C vs. HCC
46	Cluster 1	28.6704546	CDC20	C vs. HCC
45	Cluster 1	28.6704546	TOP2A	C vs. HCC
44	Cluster 1	28.6704546	ASPM	C vs. HCC
42	Cluster 1	28.6704546	AURKA	C vs. HCC
42	Cluster 1	28.6704546	CCNB2	C vs. HCC
42	Cluster 1	28.6704546	CEP55	C vs. HCC
42	Cluster 1	28.6704546	NCAPG	C vs. HCC
41	Cluster 1	28.6704546	AURKB	C vs. HCC
41	Cluster 1	28.6704546	CENPF	C vs. HCC
41	Cluster 1	28.6704546	DLGAP5	C vs. HCC
41	Cluster 1	28.6704546	KIF20A	C vs. HCC
41	Cluster 1	28.6704546	PBK	C vs. HCC
41	Cluster 1	28.6704546	TPX2	C vs. HCC
40	Cluster 1	28.6704546	HMMR	C vs. HCC
40	Cluster 1	28.6704546	NUSAP1	C vs. HCC
40	Cluster 1	28.6704546	RRM2	C vs. HCC
40	Cluster 1	28.6704546	UBE2C	C vs. HCC
39	Cluster 1	28.6704546	KIF4A	C vs. HCC
38	Cluster 1	28.8245968	FOXM1	C vs. HCC
38	Cluster 1	28.8245968	NEK2	C vs. HCC
38	Cluster 1	28.6704546	ZWINT	C vs. HCC
37	Cluster 1	28.8245968	KIF15	C vs. HCC
37	Cluster 1	28.9376344	MKI67	C vs. HCC
37	Cluster 1	28.6704546	PRC1	C vs. HCC
36	Cluster 1	28.6704546	CDKN3	C vs. HCC
36	Cluster 1	28.6704546	OIP5	C vs. HCC
36	Cluster 1	28.6704546	PTTG1	C vs. HCC
35	Cluster 1	28.6704546	HJURP	C vs. HCC
34	Cluster 1	27	CDCA5	C vs. HCC
33	Cluster 1	26.9334975	MCM4	C vs. HCC
33	Cluster 1	29	NUF2	C vs. HCC
33	Cluster 1	29	TRIP13	C vs. HCC
31	Cluster 1	27	CDC25C	C vs. HCC
30	Cluster 1	27.8193548	CENPM	C vs. HCC
25	Cluster 1	24	KIF18B	C vs. HCC
24	Cluster 2	6.22222222	IL6	C vs. HCC
16	Cluster 2	5	PTPRC	C vs. HCC
14	Cluster 2	6.22222222	CCL2	N vs. C
12	Cluster 2	6.22222222	CXCL10	N vs. C
11	Cluster 2	5.33333333	CCL19	N vs. C
11	Cluster 2	5.78571429	CCR2	C vs. HCC
10	Cluster 2	6.22222222	CXCL2	C vs. HCC
10	Cluster 2	5.78571429	LIF	C vs. HCC

### Gene Set Enrichment Analysis (GSEA)

**Fig. 4:**
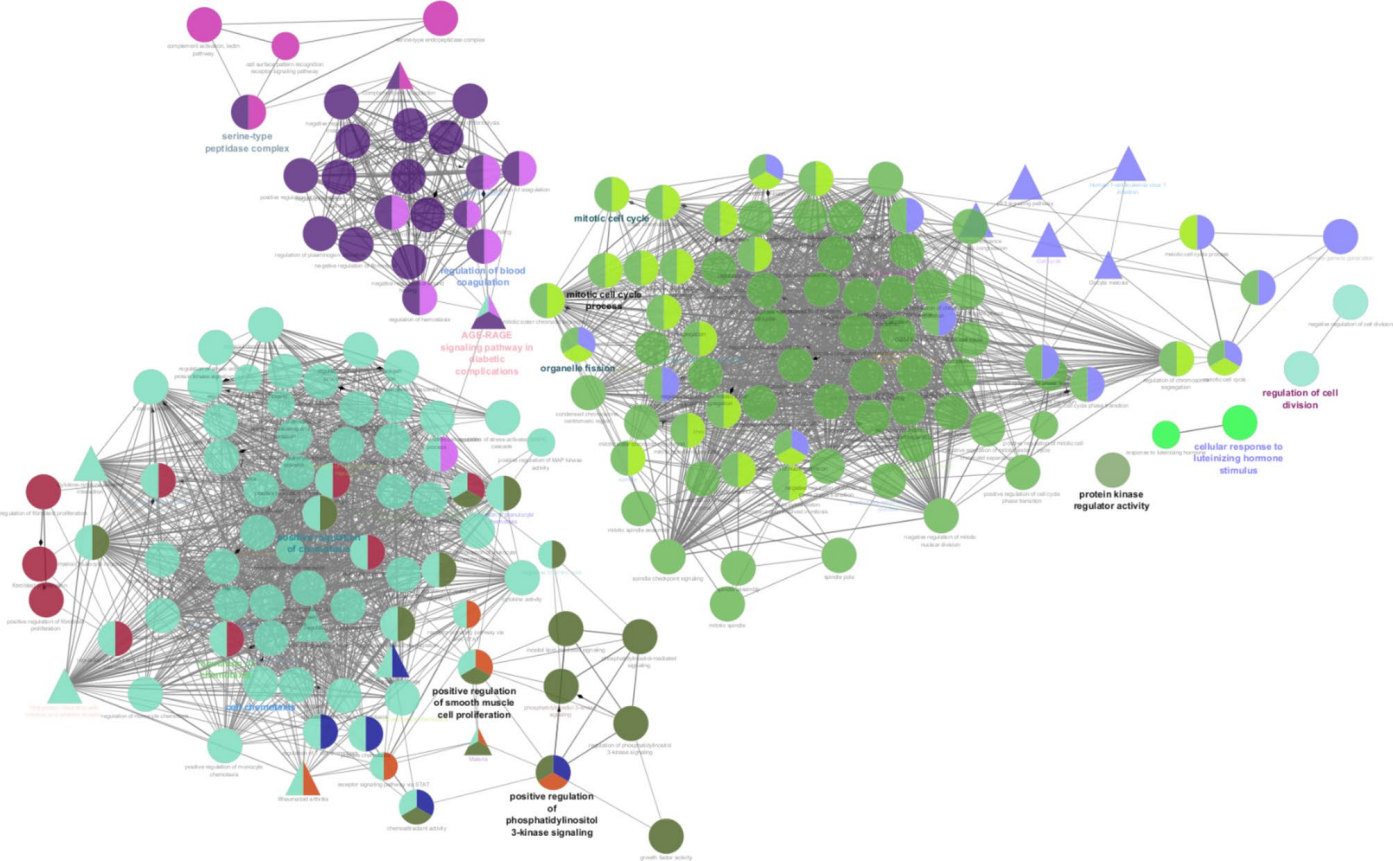
Gene Set Enrichment Analysis. Circular nodes indicated Gene ontology and triangular nodes indicate KEGG pathway.

GSEA of CCNA2 revealed their significant involvement in biological processes such as mitotic sister chromatid segregation GO:0000070, cell cycle G2/M phase transition GO:0044839, fibroblast proliferation GO:0048144 etc. KEGG pathway^1^ revealed that CCNA2 was involved in cell-cycle pathway KEGG:04110, Cellular senescence KEGG:04218 etc. (Fig. 4). Other significant genes in cluster 1 were found to be involved in chromosome separation GO:0051304, regulation of sister chromatid segregation GO:0033045 etc.

### Validation of hub genes

CCNA2 is a cell cycle regulator protein that combines with CDK2 to facilitate DNA replication when the cell enters the S phase. Moreover, it also forms the CDK1-CCNA2 complex, which is essential for cell progression into mitosis. Many malignancies exhibit overactivation of the CDK2- CCNA2 and CDK1-CCNA2 complexes, making them a promising target for cancer therapy. The disruption of CCNA2/CDK2 and CCNA2/CDK1 complexes may be facilitated by the development of highly efficient inhibitors^5,6^.

**Fig. 5:**
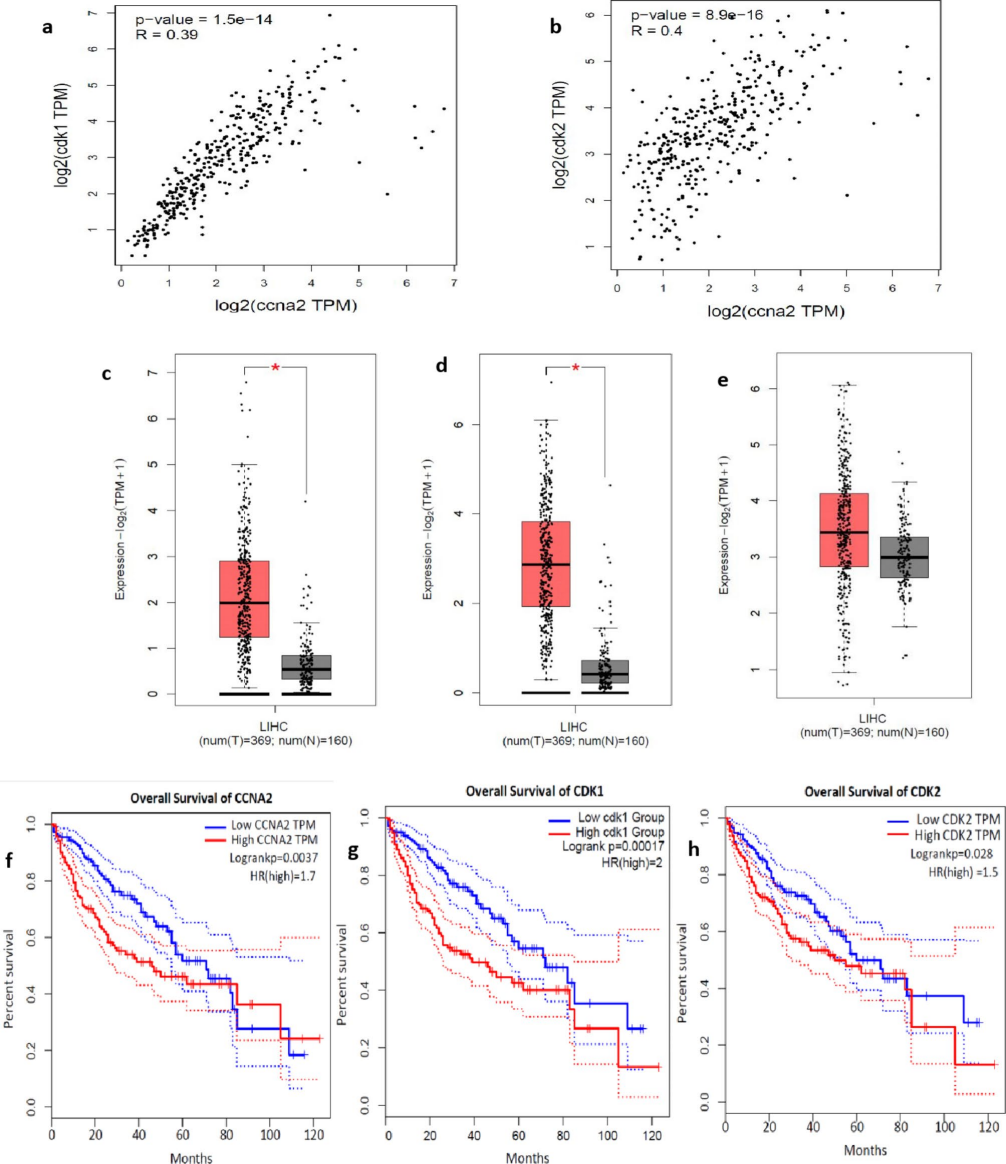
Validation of hub genes: (**a**, **b**) indicate positive correlation of expression of CCNA2- CDK1 and CCNA2-CDK2 in HCC. Fig (**c**,** d**,** e**) box plots representing the expression of CCNA2, CDK1, and CDK2 respectively in normal (grey colour) and HCC (red colour). (**f**,** g**,** h**) indicate the overall survival of patients with CCNA2, CDK1, and CDK2 expression.

Spearman’s correlation analysis of mRNA expression in GEPIA2 revealed a positive association between CDK2 and CCNA2 and between CDK1 and CCNA2 (Fig. 5a and b) in HCC. This suggests that an upregulation in the expression of CCNA2 is associated with an increased expression of both CDK2 and CDK1. Moreover, CCNA2, CDK2, and CDK1 were found to be overexpressed in HCC (Log2FC = 1 and p-value = 0.01) compared to normal (Fig. 5c, d, and e). Furthermore, high expression levels of CCNA2, CDK1, and CDK2 were significantly associated with (*p* < 0.01) poorer OS in HCC patients (Fig. 5f, g and h). Hence, CDK2 and CDK1 were chosen as the HCC targets, and a drug library was created to identify the inhibitors that may hinder the complex formation of CDKs with CCNA2, thereby inducing cell cycle arrest.

### Phase 2: creation of drug library

Given the fact that there were hitherto no FDA-approved drugs available for CDK1 and CDK2, meticulous literature mining was carried out to retrieve potential antagonists for the above targets. This search revealed Dihydroartemisinin as the tumor suppressor by targeting CDK1^7,8^; on the other hand, it demonstrated Imatinib^9^ and Bosutinib^10^ as potential antagonists against CDK2. Subsequently, a drug library was created with drugs exhibiting structural and side effect similarities with the above-mentioned standard or literature-derived drugs.

This screening process revealed ten drugs exhibiting structural similarities with Dihydroartemisinin (Table 2); however, there were no drugs that shared similar side effects. In the case of Bosutinib, 60 drugs (Table 3) were structurally similar and 335 drugs (Table 4) showed side effect similarity. Similarly, the structural similarity search of Imatinib (Table 5) yielded nine drugs, while 234 drugs shared the side effect similarity (Table 6).

**Table 2:** List of structural similar drugs of Dihydroartemisinin.

Sl No	Drug Bank ID	Similarity Score	Drugs
1	DB11638	Score 1.0	Dihydroartemisinin (standard)
2	DB06697	Score 0.991	Artemether
3	DB13851	Score 0.991	Artemotil
4	DB09274	Score 0.813	Artesunate
5	DB02772	Score 0.573	Sucrose
6	DB00581	Score 0.538	Lactulose
7	DB04465	Score 0.538	Lactose
8	DB12942	Score 0.534	Lactitol
9	DB11602	Score 0.534	Hydroxyethyl cellulose
10	DB15617	Score 0.534	Ferric derisomaltose
11	DB12434	Score 0.5	Steviolbioside

**Table 3:** List of structural similar drugs of Bosutinib.

Sl no	Drug Bank ID	Similarity Score	Drugs
1	DB06616	Score: 1.0	Bosutinib
2	DB11828	Score: 0.652	Neratinib
3	DB00317	Score: 0.596	Gefitinib
4	DB05294	Score: 0.583	Vandetanib
5	DB00530	Score: 0.554	Erlotinib
6	DB08916	Score: 0.54	Afatinib
7	DB06608	Score: 0.535	Tafenoquine
8	DB01180	Score: 0.534	Rescinnamine
9	DB01220	Score: 0.531	Rifaximin
10	DB09101	Score: 0.53	Elvitegravir
11	DB12674	Score: 0.53	Lurbinectedin
12	DB03614	Score: 0.529	Mecobalamin
13	DB06290	Score: 0.529	Simeprevir
14	DB00200	Score: 0.528	Hydroxocobalamin
15	DB00206	Score: 0.528	Reserpine
16	DB00590	Score: 0.527	Doxazosin
17	DB00696	Score: 0.526	Ergotamine
18	DB01259	Score: 0.525	Lapatinib
19	DB11963	Score: 0.524	Dacomitinib
20	DB00570	Score: 0.524	Vinblastine
21	DB00762	Score: 0.522	Irinotecan
22	DB11574	Score: 0.522	Elbasvir
23	DB01137	Score: 0.521	Levofloxacin
24	DB01165	Score: 0.521	Ofloxacin
25	DB00361	Score: 0.521	Vinorelbine
26	DB17083	Score: 0.521	Linzagolix
27	DB13783	Score: 0.52	Acemetacin
28	DB00014	Score: 0.52	Goserelin
29	DB00541	Score: 0.52	Vincristine
30	DB11613	Score: 0.52	Velpatasvir
31	DB01089	Score: 0.519	Deserpidine
32	DB00080	Score: 0.519	Daptomycin
33	DB11586	Score: 0.519	Asunaprevir
34	DB00320	Score: 0.518	Dihydroergotamine
35	DB04115	Score: 0.518	Berberine
36	DB09047	Score: 0.517	Finafloxacin
37	DB09330	Score: 0.517	Osimertinib
38	DB11800	Score: 0.516	Tivozanib
39	DB00309	Score: 0.516	Vindesine
40	DB06699	Score: 0.516	Degarelix
41	DB11641	Score: 0.516	Vinflunine
42	DB11979	Score: 0.516	Elagolix
43	DB13345	Score: 0.516	Dihydroergocristine
44	DB06719	Score: 0.515	Buserelin
45	DB11581	Score: 0.514	Venetoclax
46	DB15102	Score: 0.514	Pemigatinib
47	DB00328	Score: 0.514	Indomethacin
48	DB12141	Score: 0.513	Gilteritinib
49	DB12483	Score: 0.513	Copanlisib
50	DB01200	Score: 0.512	Bromocriptine
51	DB09078	Score: 0.512	Lenvatinib
52	DB15413	Score: 0.512	Pafolacianine
53	DB16390	Score: 0.511	Mobocertinib
54	DB09128	Score: 0.511	Brexpiprazole
55	DB01419	Score: 0.51	Antrafenine
56	DB15685	Score: 0.51	Selpercatinib
57	DB11691	Score: 0.51	Naldemedine
58	DB12010	Score: 0.51	Fostamatinib
59	DB09297	Score: 0.51	Paritaprevir
60	DB11274	Score: 0.508	Dihydro-alpha-ergocryptine

**Table 4:** List of side effect similar drugs of Bosutinib.

SI No	Drugs	SI No	Drugs	Sl No	Drugs
1	5-aminosalicylic acid	113	Colchicine	225	Mycophenolate Mofetil
2	5-Aminolevulinic Acid	114	Dipyridamole	226	Parecoxib
3	5-Aza-2’-Deoxycytidine	115	Disopyramide	227	Paroxetine
4	5-Azacytidine	116	Dobutamine	228	Auranofin
5	5-Fluorocytosine	117	Docetaxel	229	Pemetrexed
6	5-Fu	118	Diphenhydramine	230	Pantoprazole
7	6-Mercaptopurine	119	Dolasetron	231	Penciclovir
8	6-Thioguanine	120	Donepezil	232	Penicillin
9	Amlodipine Besylate	121	Gadoversetamide	233	Risperidone
10	Amoxapine	122	Galantamine	234	Ritonavir
11	Amoxicillin	123	Ganciclovir	235	Rivastigmine
12	Amphotericin B	124	Gatifloxacin	236	Rofecoxib
13	Ampicillin	125	Gefitinib	237	Ropinirole
14	Amsacrine	126	Gemcitabine	238	Ropivacaine
15	Anagrelide	127	Gemfibrozil	239	Rosiglitazone
16	Anidulafungin	128	Copolymer 1	240	Mycophenolic Acid
17	Anthracycline	129	Gemifloxacin	241	Rosuvastatin
18	Apixaban	130	Gentamicin	242	Rufinamide
19	Aprepitant	131	Glibenclamide	243	Ruxolitinib
20	Arsenic	132	Cyproterone	244	Naratriptan
21	Asenapine	133	Glipizide	245	Sitaxsentan
22	Aspirin	134	Gold Sodium Thiomalate	246	Sevoflurane
23	Atazanavir	135	Cyproterone Acetate	247	Nebivolol
24	Atenolol	136	Granisetron	248	Sibutramine
25	Atorvastatin	137	Guanidinium	249	Silver Sulfadiazine
26	Atovaquone	138	Haloperidol	250	Simvastatin
27	Axitinib	139	Cytarabine	251	Nefazodone
28	Axitinib	140	Heparin	252	Sorafenib
29	Azathioprine	141	Hepatitis B Vaccines	253	Sotalol
30	Azithromycin	142	Hexamethylmelamine	254	Sparfloxacin
31	Aztreonam	143	Histamine	255	Spironolactone
32	Belinostat	144	Hydralazine	256	Stavudine
33	Benazepril	145	Hydrochlorothiazide	257	Stiripentol
34	Benazeprilat	146	Hydrocodone	258	Streptomycin
35	Bendamustine	147	Hydroflumethiazide	259	Streptozotocin
36	Bendrofluazide	148	Hydroxychloroquine	260	Sulfadiazine
37	Benicar-Hct	149	Doxazosin	261	Olmesartan
38	Benzathine Penicillin	150	Hydroxyurea	262	Sulfamethoxazole
39	Betaxolol	151	Ibuprofen	263	Sulfasalazine
40	Bexarotene	152	D-Telaprevir	264	Nelarabine
41	Bexarotene	153	Icodextrin	265	Sulindac
42	Bezafibrate	154	Idarubicin	266	Sumatriptan
43	Bicalutamide	155	Ifosfamide	267	Sunitinib
44	Bisoprolol	156	Iloprost	268	Tacrolimus
45	Bivalirudin	157	Doxepin	269	Pentostatin
46	Bleomycin	158	Imatinib	270	Tamoxifen
47	Boceprevir	159	Imipramine	271	Irbesartan
48	Bortezomib	160	Temsirolimus	272	Telmisartan
49	Losartan	161	Indinavir	273	Temazepam
50	Bosutinib	162	Dabigatran	274	Nelfinavir
51	Bosutinib	163	Indomethacin	275	Temozolomide
52	Bupropion	164	Irbesartan	276	Teniposide
53	Busulfan	165	Isomannide	277	Terazosin
54	Cabazitaxel	166	Isoniazid	278	Terbinafine
55	Cabozantinib	167	Dabigatran Etexilate	279	Nevirapine
56	Cabozantinib	168	Isosorbide-5-Mononitrate	280	Testosterone
57	Candesartan	169	Isradipine	281	Tetracycline
58	Candesartan Cilexetil	170	Itraconazole	282	Thalidomide
59	Capecitabine	171	Ixabepilone	283	Thiazide
60	Captopril	172	Ketoconazole	284	Thioridazine
61	Ciprofloxacin	173	Dabrafenib	285	Valsartan
62	Cytarabine	174	Dacarbazine	286	Nicorandil
63	Darunavir	175	Dalbavancin	287	Nifedipine
64	Delavirdine	176	Dalteparin	288	Nilotinib
65	Diltiazem	177	Ketoprofen	289	Thiotepa
66	Doxycycline	178	Doxorubicin	290	Pentoxifylline
67	D-Penicillamine	179	Efavirenz	291	Phenylbutyric Acid
68	Duloxetine	180	Perindopril	292	Pergolide
69	Emtricitabine	181	Danazol	293	Nimodipine
70	Enfuvirtide	182	Cyclobenzaprine	294	Nabumetone
71	Eprosartan	183	Doripenem	295	Penicillin V
72	Epsilon-aminocaproic acid	184	Emtricitabine	296	Phenytoin
73	Eptifibatide	185	Entacapone	297	Pilocarpine
74	Eribulin	186	Piroxicam	298	Perindoprilat
75	Erlotinib	187	Dothiepin	299	Pentamidine
76	Estramustine	188	Pralatrexate	300	Carfilzomib
77	Estramustine	189	Ertapenem	301	Polythiazide
78	Etoposide	190	Etodolac	302	Procaine Penicillin
79	Etravirine	191	Etoricoxib	303	Promethazine
80	Famciclovir	192	Dantrolene	304	Nitric Oxide
81	Fosamprenavir	193	Dasatinib	305	Nitrofurantoin
82	Goserelin	194	Esomeprazole	306	Ponatinib
83	Irinotecan	195	Deferasirox	307	Nitrogen Mustard
84	Lisinopril	196	Ethionamide	308	Procainamide
85	Mersyndol	197	Procarbazine	309	Prochlorperazine
86	Moxifloxacin	198	Deferoxamine	310	Nitrous Oxide
87	Nilotinib	199	Delamanid	311	Nizatidine
88	Octreotide	200	Everolimus	312	Propylthiouracil
89	Pamidronate	201	Ketorolac	313	Thiothixene
90	Pazopanib	202	Delavirdine	314	Norfloxacin
91	Piperacillin	203	Ethacrynic Acid	315	Pravastatin
92	Pixantrone	204	Ethambutol	316	Pregabalin
93	Ponatinib	205	Demeclocycline	317	Nortriptyline
94	Posaconazole	206	Deprenyl	318	Ofatumumab
95	Pramipexole	207	Desipramine	319	Ofloxacin
96	Prasugrel	208	Eslicarbazepine Acetate	320	Pomalidomide
97	Propafenone	209	Digoxin	321	Paliperidone
98	Raltegravir	210	Dexamethasone	322	Olanzapine
99	Regorafenib	211	Dexmedetomidine	323	Olmesartan
100	Ribavirin	212	Dexrazoxane	324	Olsalazine
101	Rilpivirine	213	Diatrizoate	325	Omeprazole
102	Rivaroxaban	214	Diazepam	326	Oseltamivir
103	Rivastigmine	215	Diazoxide	327	Oxaliplatin
104	Sertraline	216	Glimepiride	328	Saquinavir
105	Signifor	217	Cyproheptadine	329	Naproxen
106	Som230	218	Cyclophosphamide	330	Nalidixic Acid
107	Sorafenib	219	Dichlorphenamide	331	Oxaprozin
108	Sunitinib	220	Diclofenac	332	Oxcarbazepine
109	Tenoxicam	221	Buspirone	333	Tenofovir
110	Thalidomide	222	Dicloxacillin	334	Oxprenolol
111	Thymidine	223	Didanosine	335	Oxytetracycline

**Table 5:** List of structural similar drugs of Imatinib.

Sl no	Drug Bank ID	Similarity Score	Drugs
1	DB00619	Score: 1.0	Imatinib
2	DB04868	Score: 0.751	Nilotinib
3	DB16390	Score: 0.749	Mobocertinib
4	DB09330	Score: 0.603	Osimertinib
5	DB12001	Score: 0.602	Abemaciclib
6	DB14726	Score: 0.519	Dabigatran
7	DB11697	Score: 0.509	Pacritinib
8	DB08901	Score: 0.51	Ponatinib
9	DB06695	Score: 0.505	Dabigatran etexilate

**Table 6:** List of side effect similar drugs of Imatinib.

Sl No	Drugs	Sl No	Drugs	Sl No	Drugs
1	Aliskiren	79	Samarium	157	Nilotinib
2	Ambrisentan	80	Saquinavir	158	Ofloxacin
3	Aprepitant	81	Sitaxsentan	159	Oxaliplatin
4	Aripiprazole	82	Sofosbuvir	160	Paclitaxel
5	Atazanavir	83	Sorafenib	161	Pazopanib
6	Atropine	84	Stavudine	162	Pemetrexed
7	Axitinib	85	Sunitinib	163	Pentamidine
8	Azilsartan	86	Telmisartan	164	Pimecrolimus
9	Azithromycin	87	Temsirolimus	165	Piroxicam
10	Bendamustine	88	Tenofovir Disoproxil Fumarate	166	Podophyllotoxin
11	Bicalutamide	89	Tinidazole	167	Ponatinib
12	Bivalirudin	90	Pemetrexed	168	Indomethacin
13	Bosentan	91	Tirofiban	169	Pramipexole
14	Bromazepam	92	Tramadol	170	Raltitrexed
15	Caffeine	93	Trandolapril	171	Retinoic Acid
16	Cancidas	94	Posaconazole	172	Ixabepilone
17	Capecitabine	95	Trovafloxacin	173	Ribavirin
18	Carbidopa	96	Vandetanib	174	Risperidone
19	Cefaclor	97	Warfarin	175	Rivastigmine
20	Cefditoren	98	5-Aminolevulinic Acid	176	Ropinirole
21	Cefpodoxime	99	8-MOP	177	Sertaconazole
22	Cefuroxime	100	Cancidas	178	Sorafenib
23	Celecoxib	101	Ge-132	179	Sumatriptan: Rare
24	Citalopram	102	Acitretin	180	Tamoxifen
25	Clarithromycin	103	Acyclovir	181	Tamsulosin
26	Clopidogrel	104	Adapalene	182	Tazarotene
27	Crizotinib	105	Allopurinol	183	Temozolomide
28	Dabigatran Etexilate	106	Amphotericin B	184	Terbinafine
29	Dexmedetomidine	107	Arsenic	185	Thalidomide
30	Dexrazoxane	108	Arsenic Trioxide	186	Thiotepa
31	Diazoxide	109	Atovaquone	187	Vinflunine
32	Disopyramide	110	Benzyl Alcohol	188	Zalcitabine
33	Docetaxel	111	Bleomycin Hydrochloride	189	Ziconotide
34	Emtricitabine	112	Busulfan	190	K779
35	Ertapenem	113	Capecitabine	191	PEP005
36	Eslicarbazepine Acetate	114	Ceftazidime	192	Acetaminophen
37	Etravirine	115	Cevimeline	193	Aliskiren
38	Everolimus	116	Chlorhexidine	194	Betaxolol
39	FAMP	117	Propafenone	195	Lidocaine
40	Febuxostat	118	Chlorhexidine Gluconate	196	Brinzolamide
41	Fentanyl	119	Chloroquine	197	Cabergoline
42	Fludarabine	120	Ciclopirox	198	Cetirizine
43	Fondaparinux	121	Clindamycin	199	Clonazepam
44	Fosaprepitant Dimeglumine	122	Clobetasol	200	Clozapine
45	Fosfomycin	123	Clofarabine	201	Cytokinin
46	Fosfomycin Trometamol	124	Cytarabine	202	Deferiprone
47	Gadobenate Dimeglumine	125	Dasatinib	203	Dihydroergotamine
48	Gadoversetamide	126	Delavirdine	204	Diltiazem
49	Gemifloxacin	127	Desonide	205	Donepezil
50	Ibuprofen	128	Dexamethasone Sodium Phosphate	206	Doxycycline
51	Iloperidone	129	Diclofenac	207	Famciclovir
52	Imatinib	130	Docetaxel	208	Fusidic Acid
53	Imiquimod	131	Enoxaparin	209	Hetastarch
54	Indinavir	132	Erlotinib	210	Imatinib
55	Lansoprazole	133	Ertapenem	211	Iodixanol
56	Lapatinib	134	Erythromycin	212	Iopamidol
57	Lenalidomide	135	Esmolol	213	Ioversol
58	Lercanidipine	136	Estramustine	214	Latanoprost
59	Leuprorelin Acetate	137	Estramustine Phosphate	215	Methsuximide
60	Levodopa/Carbidopa	138	Etodolac	216	Nifedipine
61	Levonorgestrel	139	Everolimus	217	Pimozide
62	Levosimendan	140	Furosemide	218	Risperidone
63	Meropenem	141	Flurbiprofen	219	Salmon Calcitonin Acetate
64	Meropenem Anhydrous	142	Quetiapine	220	Linagliptin
65	Methylene Blue	143	Gabapentin	221	Sertraline
66	Mitoxantrone	144	Gemcitabine	222	Sulfadiazine
67	Moxifloxacin	145	Gemifloxacin	223	Sulfasalazine
68	N-Carbamylglutamate	146	Ribavirin	224	Lomefloxacin
69	Nelfinavir	147	Guanfacine	225	Sunitinib
70	Nilotinib	148	Hydroxyurea	226	Topiramate:
71	Nitrofurantoin	149	Icodextrin	227	Travoprost
72	Olanzapine	150	Imatinib	228	Zolpidem
73	PCI-32,765	151	Rifampicin	229	Memantine
74	Photofrin II	152	Rilpivirine	230	Methyl Aminolevulinate
75	Refludan	153	Riluzole	231	Methylphenidate
76	Revasc	154	Risperidone	232	Metronidazole
77	Losartan	155	Ropinirole	233	Nevirapine
78	Triptorelin	156	Rotigotine	234	Niacin

## Phase 3: In-silico analysis

### Screening of drug library

PDB was explored to retrieve X-ray crystallographic structures of CDK1. This search yielded 182 structures, among them 181 corresponded to “Homosapiens”. As this study is focused on CDK1 antagonists the PDBs with co-crystallized compounds with agonistic properties were excluded. The PDB-4Y72 that displayed CDK1 bound with inhibitor{[(2,6-difluorophenyl) carbonyl] amino}-N-(4-fluorophenyl)-1 H-pyrazole-3-carboxamide was chosen for the study. The interactive patterns at the binding interface between the co-crystallized inhibitor and CDK1 were retrieved to identify the crucial residues. Residues were selected for receptor grid generation based on the interactive profile. The created drug libraries were then screened for their affinity towards CDK1 binding sites.

Of the 607 X-ray crystallographic structures of CDK2 identified from PDB, 588 were associated with “Homosapiens”; however, agonistic co-crystallized molecules were rejected. Subsequently, PDB- 2FVD, with diaminopyrimidine inhibitor as co-crystallized ligand, was chosen for the investigation. The binding interface between the diaminopyrimidine inhibitor/CDK2 complex was scrutinized to detect key residues, and a receptor grid was created using the interactive profile. The created drug libraries were then screened for their affinity towards the CDK2 binding site.

XP screening of the Dihydroartemisinin drug library yielded 4 molecules with a docking score ranging from − 9.151 to -3.357. Further, these drugs were subjected to MMGBSA and Qikpro analysis. Among these shortlisted drugs, Steviolbioside exhibited better interaction, with no CNS permeability and no violations in Lipinski rule of five, and was further processed for molecular dynamics studies along with Dihydroartemisinin and co-crystallized CDK1 inhibitor (Table 7).

**Table 7:** Molecular docking parameters, binding energy, and pharmacokinetic properties of the selected drugs pertinent to CDK1 and CDK2.

Compounds	Targets	Docking score	CNS	Rule of Five	MMGBSA dG Bind(NS)
Steviolbioside	CDK1	-9.151	0	0	-50.096
Dihydroartemisinin		-4.09	0	0	-43.2066
Valsartan	CDK2	-6.63	-2	0	-60.31
Irbesartan		-6.644	-2	0	-51.69
Losartan		-7.601	-2	0	-65.93
Olmesartan		-7.381	-2	0	-63.23
Fludarabine		-7.365	-2	0	-46.55
Nitrofurantoin		-6.652	-2	0	-35.7
Lisinopril		-8.659	-2	0	-50.04
Furosemide		-8.189	-2	0	-52.07
Etravirine		-6.836	-2	0	-52.09
Enoxaparin		-12.536	-2	2	-54.24
Dobutamine		-8.875	-2	0	-57.27
Allopurinol		-6.219	-1	0	-26.32
Imatinib		-10.330	-1	0	-61.87
Aspirin		-6.425	-1	0	-24.09
Bosutinib		-4.914	1	1	-30.43

HTVS of the CDK2 library yielded 600 drug molecules with docking scores ranging from − 7.613 to -1.368 and glide energy from − 58.902 to -2.478 kcal/mol. Further screening through SP mode resulted in 472 compounds with docking scores ranging from − 8.9 to -2.25 and glide energy ranging between − 60.73 to -27.59. Then, screening through XP mode yielded 366 compounds with docking scores ranging from − 12.536 to -2.162 and glide energy from − 85.585 to -36.86. Herein, standard drugs such as Bosutinib and Imatinib exhibited a docking score of -4.914 and − 10.330, respectively. The compounds with docking scores more than − 4.914 were shortlisted and subjected to MMGBSA and qikpro analysis. Among these shortlisted drugs, only 105 exhibited no CNS permeability with no violations of Lipinski’s rule of five. Among 105 drugs, 12 drugs that were not explored for their interactions with CDK2 from both Imatinib and Bosutinib libraries along with the standard drugs (Imatinib and Bosutinib) and Diaminopyrimidine (Co-crystallized ligand of CDK2 - PDB- 2FVD) were further processed for molecular dynamics (Table 7).

### Molecular dynamics

Post docking, the drugs were shortlisted for molecular dynamics analysis based on the following parameters: (1) drugs that demonstrated no literature support pertaining to their interaction with CDK1 and/or CDK2, (2) drugs that exhibited substantial interactions with binding pockets in terms of low binding free energy, specificity/best fit within the pocket, and (3) the drugs that showcased favorable pharmacokinetic properties such as CNS non-permeability, and compliance with the rule of five. Herein, Steviolbioside, from the Dihydroartemisinin library, abided by the set criteria with respect to CDK1 and hence entered the molecular dynamics phase and was compared to the standard drug. Similarly Dobutamine, Irbesartan, Lisinopril, Losartan, Nitrofurantoin, Olmesartan and Valsartan, belonging to the Bosutinib library; Allopurinol, Fludarabine, Nitrofurantoin, Lisinopril, Losartan, Furosemide, Etravirine, Enoxaparin and belonging to Imatinib library; displayed compliance towards the pick out criteria concerning CDK2 complex and thus were processed for molecular dynamics with their respective standards.

**Fig. 6:**
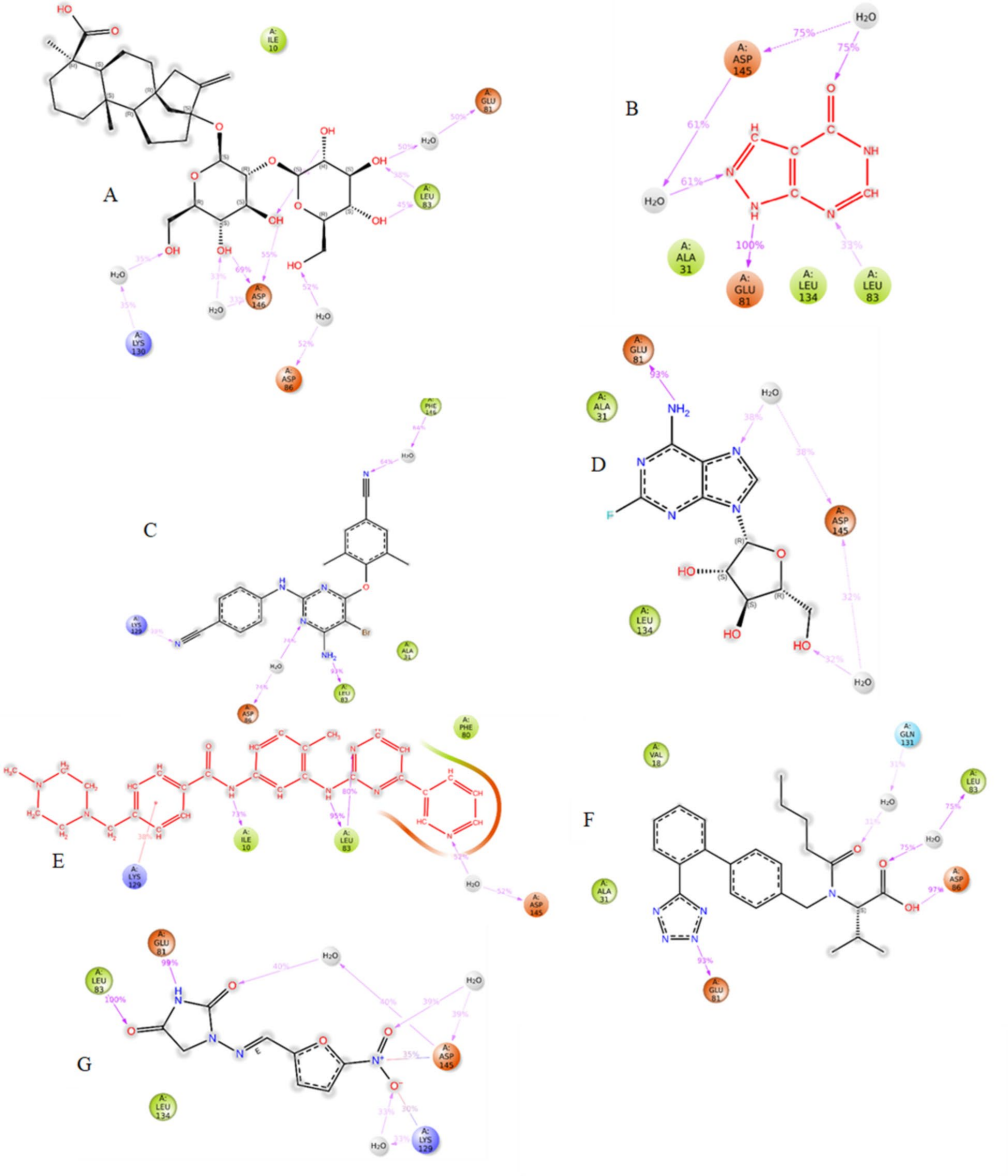
2D Illustration of protein-ligand complex (**A**) CDK1-Steviolbioside, (**B**) CDK2-Allopurinol, (**C**) CDK2-Etravirine, (**D**) CDK2-Fludarabine, (**E**) CDK2-Imatinib, (**F**) CDK2-Losartan, (**G**) CDK2-Nitrofurantoin.

**Fig. 7:**
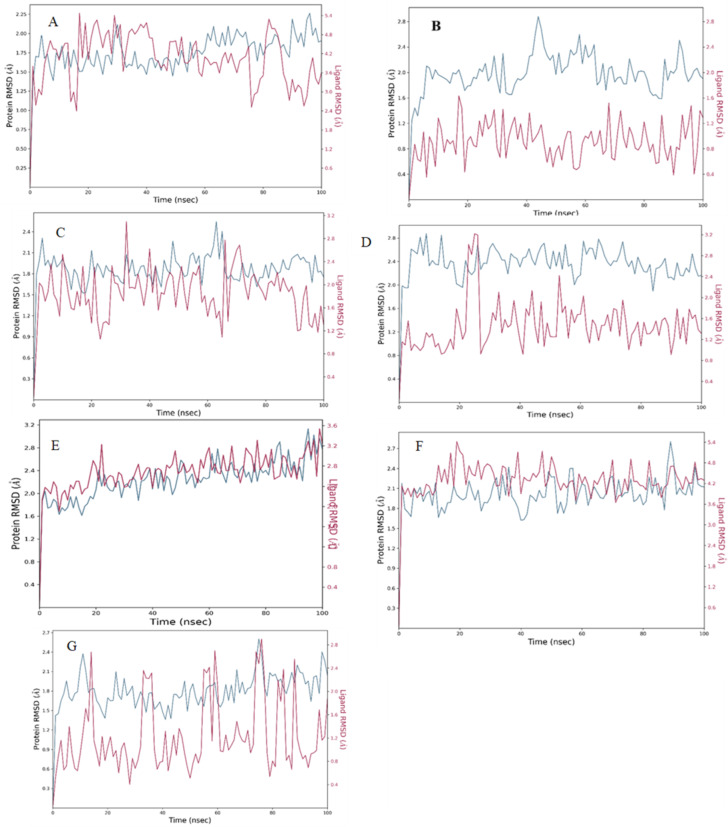
Simulation plot of Root Mean Square Deviation showing protein (red) and ligand (blue). (**A**) CDK1-Steviolbioside, (**B**) CDK2-Allopurinol, (**C**) CDK2-Etravirine, (**D**) CDK2-Fludarabine, (**E**) CDK2-Imatinib, (**F**) CDK2-Losartan (**G**) CDK2-Nitrofurantoin.

**Fig. 8:**
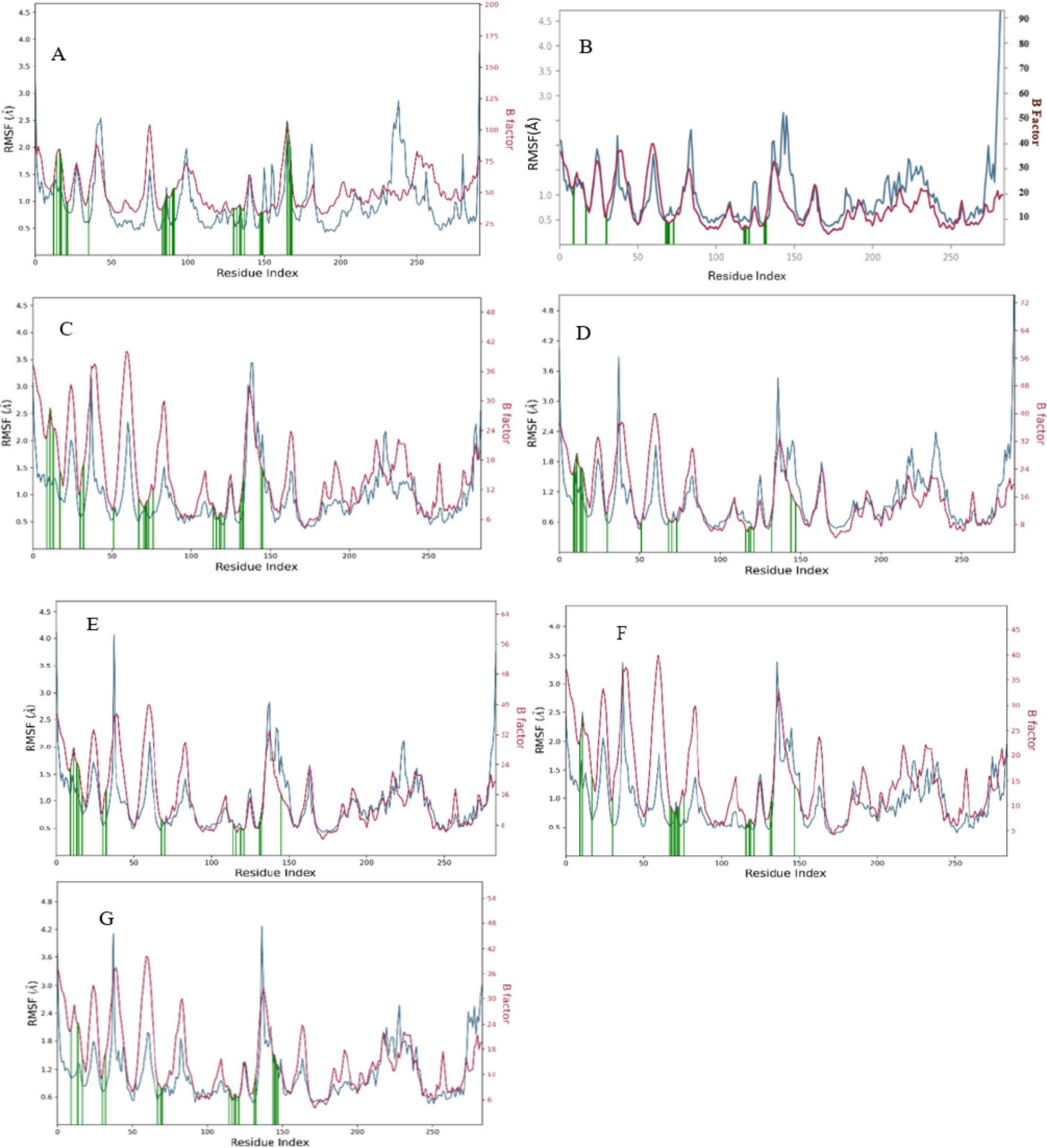
Simulation plot showing protein RMSF (blue), ligand contact (green), and B factor (red). (**A**) CDK1-Steviolbioside, (**B**) CDK2-Allopurinol, (**C**) CDK2-Etravirine, (**D**) CDK2-Fludarabine, (**E**) CDK2-Imatinib, (**F**) CDK2-Losartan (**G**) CDK2-Nitrofurantoin.

**Fig. 9:**
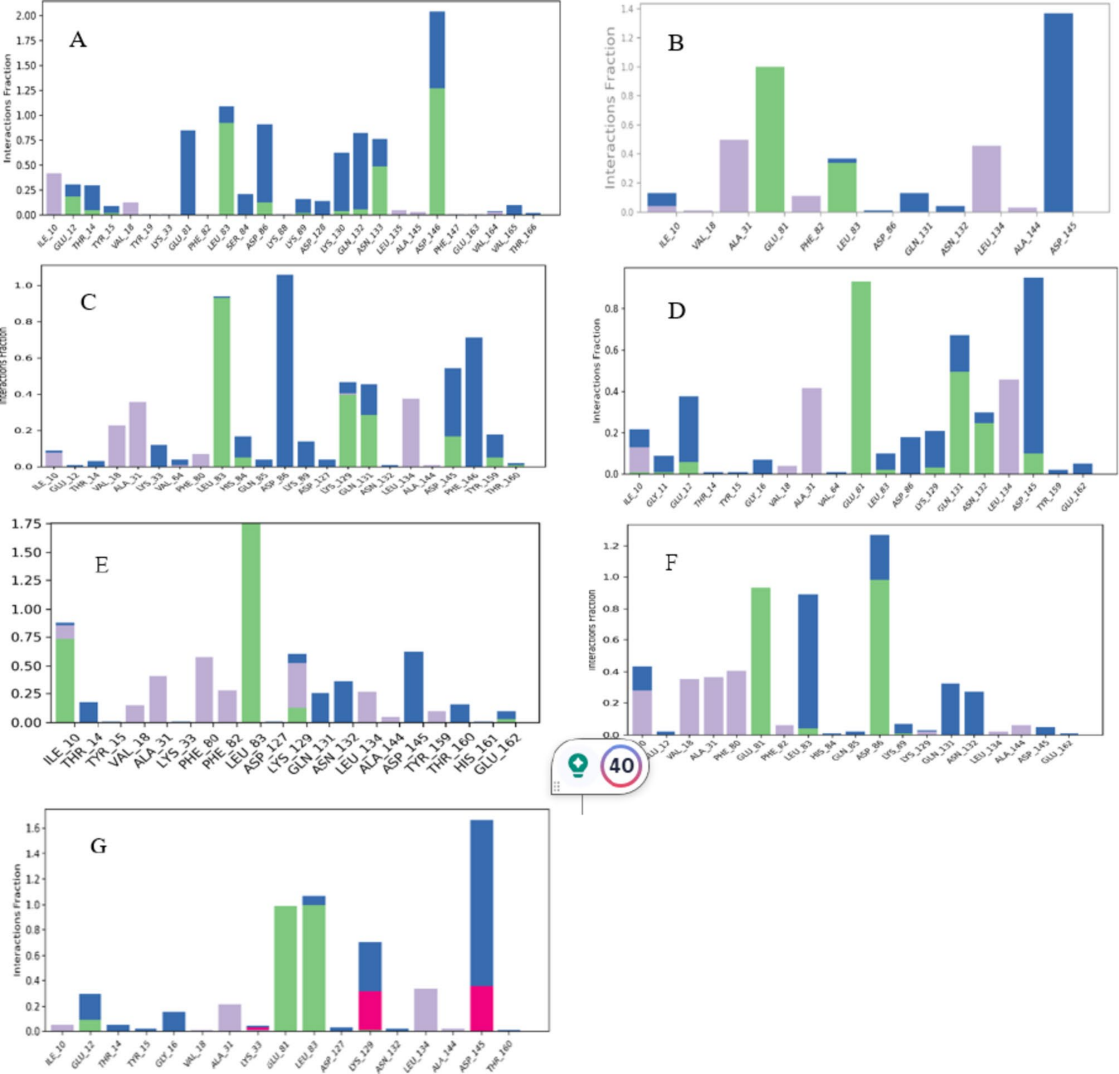
Illustration of protein-ligand contact map. (**A**) CDK1-Steviolbioside, (**B**) CDK2-Allopurinol, (**C**) CDK2-Etravirine, (**D**) CDK2-Fludarabine, (**E**) CDK2-Imatinib, (**F**) CDK2-Losartan (**G**) CDK2-Nitrofurantoin.

RMSD of Steviolbioside exhibited substantial instability within the CDK1 binding pocket owing to its high RMSD between 3.0 A⁰ to 5.4 A⁰ throughout the simulation, which was much higher than the protein RMSD. It displayed four contacts, and the stability of hydrogen bonds and water bridges with ASP146 remained consistent throughout the simulation; however, the higher RMSD indicated instability of the drug-ligand complex. Hence, Steviolbioside was disqualified for further in vitro studies (Figs. 6, 7, 8 and 9).

Out of the candidates that were shortlisted with respect to CDK2 ligand complex, drugs such as Allopurinol, Etravirine, Fludarabine, Imatinib, Losartan and Nitrofurantoin were observed to exhibit favorable stability within the binding pocket when compared to their respective standard Imatinib. These compounds demonstrated minimal fluctuations in the protein backbone and maintained stable interactions with essential residues, particularly in the 80–89 region of the ATP binding pocket of the CDK2 (Figs. 6, 7, 8 and 9). Post-MM/GBSA analysis was conducted for drugs such as Etravirine(− 57.03 Kcal/mol), Fludarabine(− 44.17.06 Kcal/mol), Losartan (− 56.06 Kcal/mol), Imatinib (− 69.49 Kcal/mol), and Nitrofurantoin (− 41.89 Kcal/mol), demonstrating enhanced dynamic results, which were subsequently prioritized for in-vitro cytotoxic assays.

## Phase 3: In-vitro analysis

### Cytotoxicity assay

**Fig. 10:**
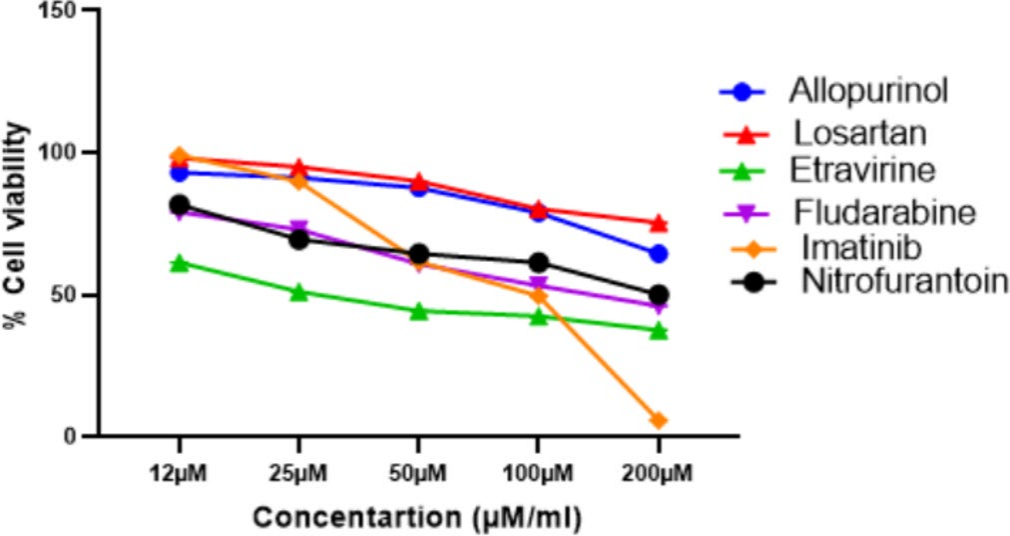
Cytotoxic assay of drugs in Huh7 cell lines.Values are expressed as mean ± SD.

The cytotoxicity of the selected repurposable drugs was assessed in Huh-7 cell lines. Herein, Etravirine and Imatinib demonstrated significant cytotoxicity at 36.41µM/ml and 60.37µM/ml, respectively. Conversely, Allopurinol (164.35 µM/ml), Fludarabine (119.94 µM/ml), and Nitrofurantoin (226.75 µM/ml) exhibited moderate cytotoxicity. Losartan displayed a high IC_50_ value of 326.63 µM/ml, indicating low cytotoxicity (Fig. 10).

Consequently, based on the significant cytotoxicity compared to other drugs, Etravirine, Fludarabine, and Imatinib were considered for further investigations.

### CDK-2 assay

**Fig. 11:**
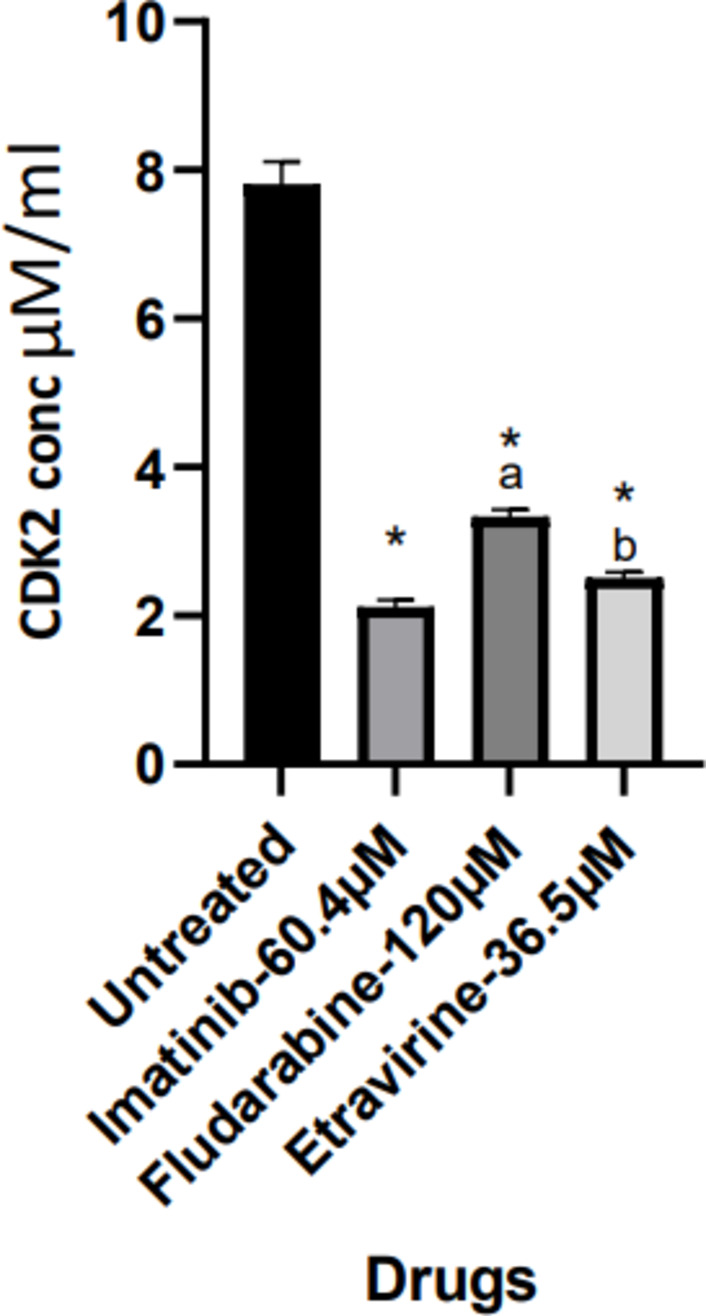
CDK2 inhibitory activity of Etravirine, Fludarabine, and Imatinib in Huh-7 cells. Values are expressed as mean ± SD (*n* = 3). ap < 0.001 and bp < 0.01 Vs standard Imatinib; *p < 0.001 significant Vs untreated group. Data was analyzed by one-way ANOVA followed by Tukey-Kramer multiple comparison test.

Imatinib, Fludarabine, and Etravirine demonstrated substantial cellular CDK2 inhibition at their respective IC_50_ concentrations. Imatinib inhibited CDK2 more effectively than Fludarabine and Etravirine; however, Etravirine showed inhibition analogous to Imatinib as compared to Fludarabine. Nevertheless, further studies were performed for deeper comprehension of the molecular processes underlying these compounds’ anti-cancer efficacy (Fig. 11).

### CDK2 and CCNA2 expression analysis

**Fig. 12:**
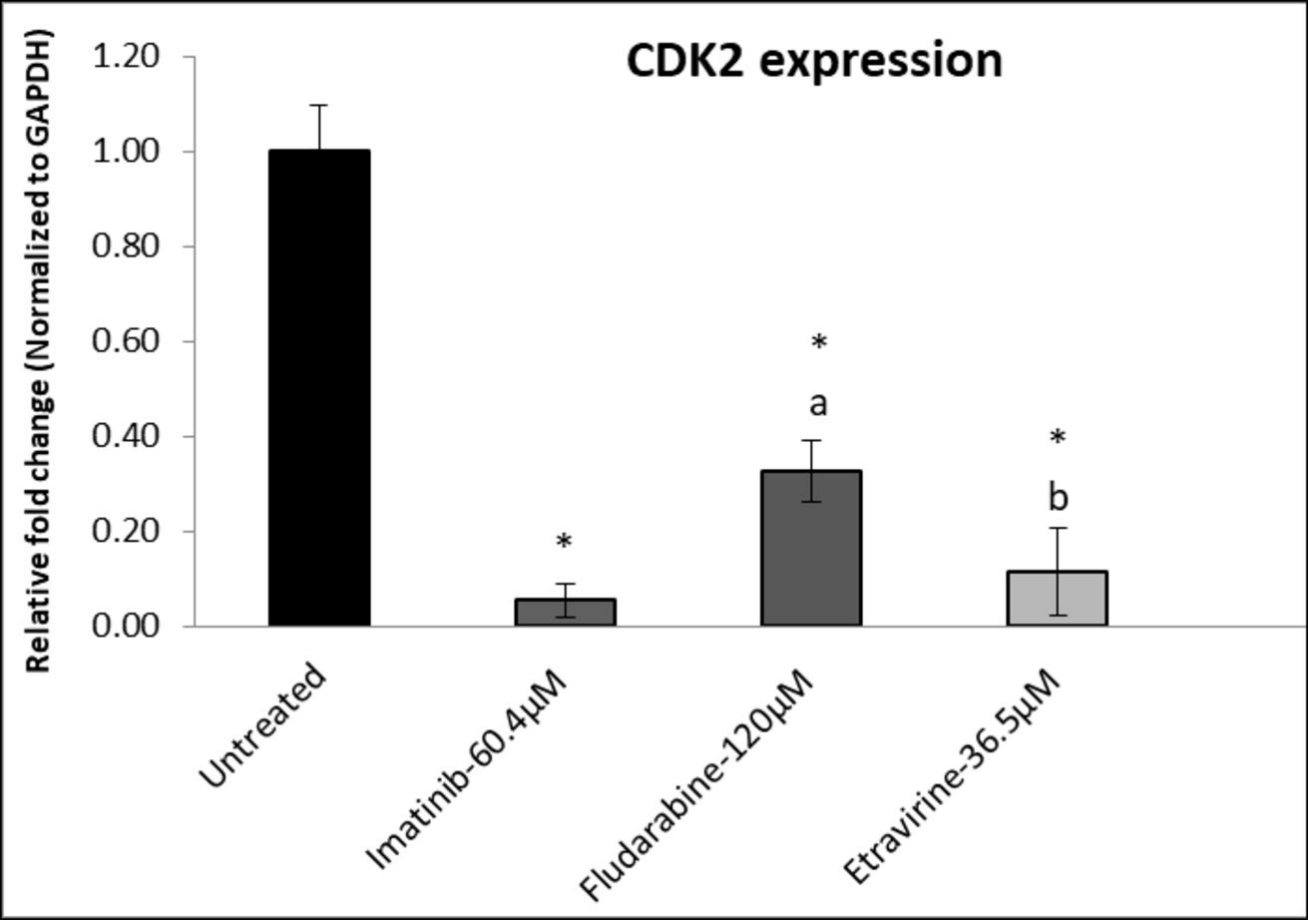
Effect of Imatinib, Fludarabine, and Etravirine on CDK2. Values are expressed as mean ± SD (*n* = 3). ^a^*p*<0.001 and ^b^*p*<0.01 Vs standard Imatinib; *p < 0.001 Vs untreated group. Data was analyzed using one-way ANOVA followed by a Tukey-Kramer multiple comparison test.

**Fig. 13:**
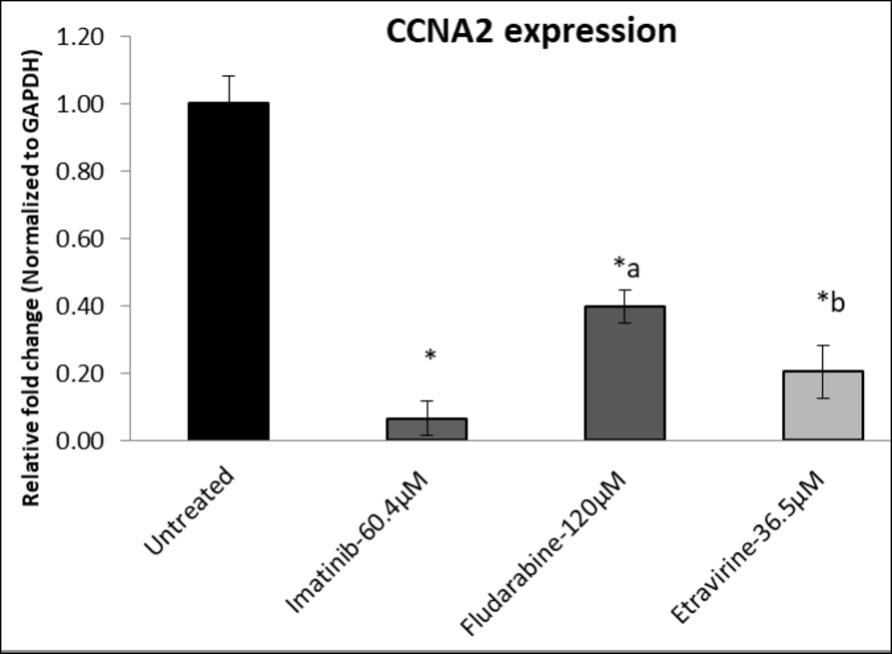
Effect of Etravirine, Fludarabine, and Imatinib on CCNA2. Values are expressed as mean ± SD (*n* = 3). ^a^*p*<0.001 and ^b^*p*<0.01 *Vs* standard Imatinib; *p < 0.001 *Vs* untreated group. Data was analyzed using one-way ANOVA followed by a Tukey-Kramer multiple comparison test.

The untreated Huh7 cells showed a relative Log Fold Change (Log FC) value of 1 for the expression of CDK2 and CCNA2. Upon treatment with IC_50_ concentration of selected drugs, there was an overall significant decrease in the expression of both CDK2 and CCNA2. Imatinib, significantly decreased the relative Log FC value of CDK2 and CCNA2 to 0.056 ± 0.01 and 0.07 ± 0.02, respectively. Fludarabine treatment resulted in reduced expression of CDK2 and CCNA2, with a Log FC value of 0.33 ± 0.11 and 0.40 ± 0.08, respectively. Etravirine treatment substantially decreased CDK2 and CCNA2 expression, with a Log FC value of 0.12 ± 0.07 and 0.21 ± 0.14, respectively (Figs. 12 and 13).

Imatinib showed a noteworthy decrease in the Log FC values of CCNA2 and CDK2, indicating a substantial downregulation of these critical cell cycle regulators. Similarly, Fludarabine and Etravirine treatment, also resulted in decreased expression of CDK2 and CCNA2. These findings emphasized the need for a comprehensive understanding of the cellular responses induced by Imatinib and Etravirine, particularly in modulating the cell cycle dynamics.

### Cell cycle analysis

**Fig. 14:**
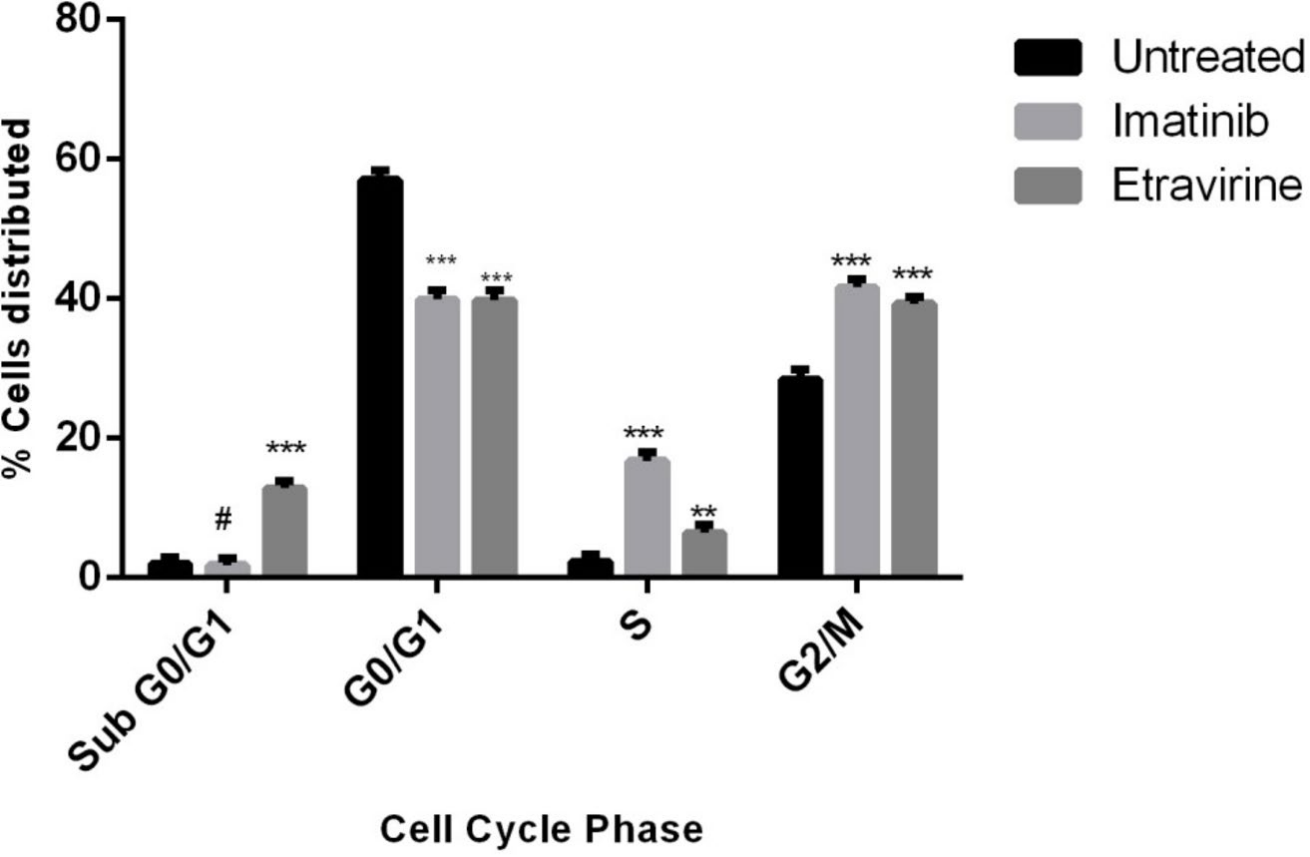
Percentage cell distributed in the different phases of Huh-7 cell cycle upon treatment with Imatinib and Etravirine in comparison to the control (Untreated). Values are expressed as mean ± SD (*n* = 3). **p < 0.01, ***p < 0.05, #non-significant *Vs* untreated group. Data was analyzed by one-way ANOVA followed by Tukey-Kramer multiple comparison test.

The impact of Etravirine and Imatinib in different phases of cell cycle in Huh-7 cells was analyzed by flow cytometry. At their respective IC50 concentrations, Imatinib and Etravirine-treated cell lines showed cell cycle arrest in 1.06%, and 13.04% of cells in the Sub G0/G1 phase associated with apoptosis when compared to 1.38% observed in untreated. The apoptotic rates in the G0/G1 phase (the growth phase) of cells treated with Imatinib and Etravirine were 41% and 40.66%, respectively, compared to 68.51% in the untreated group. In the S phase (synthetic phase), 16.21% and 7.17% of cells were arrested in the Imatinib and Etravirine-treated groups, respectively, in contrast to 1.76% in the untreated group. In the G2/M phase, 28.35%, 41.73%, and 39.13% of cells were halted in the untreated, Imatinib-treated, and Etravirine-treated groups, respectively (Fig. 14).

## Discussion

Drug repositioning, a strategy that unveils the repurposing potential of FDA-approved drugs for a new indication, has drawn the interest of pharmaceutical companies and medical investigators, as the denovo drug discovery pipeline often encounters inevitable challenges in terms of high attrition rates alongside modest efficacy and untoward toxicity profile. As the safety profile of the drugs mostly chosen for repurposing is well established, this approach might be a panacea for conditions, especially with potentially fatal consequences, and the existing approved therapeutic agents are extremely toxic. Malignancy disorder is one such condition with unmet medical needs, particularly in the case of cancers with high mortality rates, such as HCC and pancreatic cancer. The invasive nature of the illness and the lack of an effective, individualized treatment strategy demand a heightened focus on the discovery of new drugs or drug combinations with tolerable safety profiles to confront HCC.

It is paramount to comprehend the intricate pathogenic mechanisms that contribute to tumor heterogeneity in order to appraise the pace of cancer progression. Although there are a plethora of research reports on the etiological mediators and cellular-level molecular aberrations in HCC, the role of crucial genes that are differentially expressed and their respective protein interactions with other established key proteins of the pathological cascades of the tumour milieu is however unclear. Hence, a substantial dearth of target-guided drug discovery results in inadequate therapeutic strategies with trivial translational potential.

This study aimed to unlock the repurposable potential of FDA-approved drugs against disease- specific genetic targets governing the pathological pathways of HCC. Herein, transcriptomics microarray datasets were explored to capture the precise genetic landscape that crucially partakes the developmental stages of HCC as a strategy to unveil the potential targets for drug repurposing. At the outset, the DEGs that contribute to the significant progression of HCC were discerned from microarray data comprising normal vs. cirrhosis and cirrhosis vs. HCC by leveraging appropriate bioinformatics tools. Subsequently, the PPI network was constructed using STRING to elucidate the interactive potential of the proteins that are coded by the shortlisted HCC-specific DEGs, along with their intricate molecular mechanisms. The application of the MCODE algorithm at this juncture aided in grouping 86 highly interconnected proteins into 12 clusters. Notably, among 37 proteins in cluster 1, CCNA2, upregulated in HCC, showed the highest interaction with other co- expressed proteins and hence was considered the potential cross-talk gene.

CCNA2, a cell cycle regulator, activates CDK1/CDK2 by forming a functional heterodimeric complex, which is crucial for triggering the cell cycle progression. Overexpression of CCNA2, along with CDK1 and CDK2, is seen in a number of malignancies, including HCC. This finding was confirmed by mRNA expression analysis in GEPIA2, which showed a strong positive correlation of CCNA2 with CDK1 (P value = 1.5e-14) as well as CDK2 (8.9e-16) in HCC. Moreover, CCNA2, CDK1, and CDK2 mRNA expression were significantly higher in HCC than in normal hepatic tissues. Similar to the GO of CCNA2, other high node degree proteins in cluster 1, such as AURKA, AURKB, CCNB1, CDC25, CENPF, FOXM1, and SKP2, were involved in the G2/M transition of the mitotic cell cycle which is on par with KEGG pathway result from Clue Go^11–13^. Dysregulation of these genes promotes tumor growth and metastasis. Furthermore, DEGs (cluster 1) such as AURKB, CCNB1, CDC20, CENPF, NUF2, PBK, TPR, TRIP13, and ZWINT were also involved in cell cycle checkpoint signaling.

Generally, during irreparable DNA damage, the aforementioned checkpoint signal regulators hamper the cell cycle progression, resulting in apoptosis. Cancer-related mutations that manipulate the cell cycle regulators result in unabated cell division, largely by reducing the ability of aberrantly regulated cells to exit the cell cycle. Additionally, Clue Go analysis revealed that CCNA2 and its co-expressed DEGs significantly enriched the P53 signaling pathway, cell cycle, and chemokine signaling pathway. CCNA2 mediates its action by interacting with CDK1 and CDK2; hence, these kinases were chosen as the HCC targets.

A drug library was created to collate the inhibitors that may hinder the complex formation of the kinases with CCNA2, thereby inducing cell cycle arrest. Since both the targets lacked approved standard inhibitors, the drug repository was established by a thorough literature mining to capture literature-derived inhibitors for CDK1 and CDK2. This exposed the tumor-suppressing properties of Dihydroartemisinin by its interaction with CDK1 and was considered standard for creating drug library^14^. Similarly, Imatinib and Bosutinib emerged as notable antagonists of CDK2 and consequently underwent systematic inclusion in the construction of drug repositories for CDK2. Further, based on the concept that drugs with structural and side effect similarities might exhibit action by sharing common therapeutic targets, the drug libraries were created for the above standards. Following this, the drugs from the libraries were screened against their respective targets via molecular docking tailed by molecular dynamics studies.

The molecular docking studies of the Dihydroartemisinin library revealed only Steviolbioside to be a potential drug candidate against CDK1 based on its interaction with the ATP binding pocket, which is comparable with the interactions of ATP-competitive inhibitors. This binding pocket is characterized by a “DFG” motif containing ASP146, which plays a critical role in facilitating phosphorylation reactions by binding with ATP. Even though Steviolbioside showed a substantial interaction with the ASP146 residue, molecular dynamics analysis for this ligand was not promising, as its RMSD with CDK1 displayed a higher degree of fluctuation, indicating the instability of ligand-protein complex.

Similarly, docking results showed that 15 drugs from Imatinib and Bosutinib libraries interacted with CDK2 residues comparable to those of ATP-competitive inhibitors by acting on amino acid residues near the ATP binding site. The interactions in the hinge region (composed of residues 80–84) that link the N- and C-terminal lobes of CDK2 are an integral part of the ATP adenine binding site^15^. Herein, drugs such as Etravirine, Fludarabine, Nitrofurantoin, Irbesartan, Valsartan, Lisinopril, and Allopurinol exhibited interaction with residues 80–84. Also, Furosemide, Enoxaparin, Dobutamine, Nitrofurantoin, Lisinopril, Allopurinol, and Imatinib interacted with ASP 145 in the “DFG motif,” which plays a vital role in kinase activity. Moreover, Etravirine, Enoxaparin, and Valsartan also interacted with ASP86, a critical ATP binding pocket residue, whose mutation may impact ATP binding and, therefore, interfere with CDK2 activity^16^.

A comprehensive molecular dynamics study was performed for a set of 16 drugs. Among these, one drug was sourced from the Dihydroartemisinin library, while the remaining fifteen were depicted from the Imatinib and Bosutinib libraries. Drugs from Imatinib library, such as Losartan, Etravirine, Fludarabine, Nitrofurantoin, Allopurinol, and Imatinib, exhibited better interaction with CDK2 without protein backbone distortion. As a crucial step in bridging computational predictions with biological responses, these drugs were further subjected to the in vitro validation phase in Huh-7 cell lines.

Research indicates that CDK2 plays a pivotal role in regulating the proliferation of adult hepatocytes in response to acute mitogenic signals. Furthermore, CDK2 is crucial for cyclin D1-mediated centrosome overduplication, which impacts to chromosomal instability and supports tumor progression^17^. Additionally, studies have shown that the concurrent overexpression of cyclin E and CDK2 in HCC is associated with enhanced proliferative activity in cancerous hepatocytes^18^. These findings collectively reinforce the potential of CDK2 as a promising therapeutic target for HCC.

Initially, the MTT assay assessed the cytotoxic potential of the shortlisted drugs. Nitrofurantoin, Fludarabine, and Allopurinol at a concentration of 12.5 µM/ml demonstrated cytotoxicity in the Huh-7 cell line comparable to the standard Imatinib. Similarly, at a concentration of 100 µM/ml, there was no distinguished variation in the cytotoxic impact of Etravirine and Imatinib. Etravirine and Fludarabine, which showed significant cytotoxicity in low IC_50_ value, were shortlisted for further studies.

The results of the CDK2 assay revealed a well-pronounced inhibitory potential of Imatinib, and Etravirine when compared to Fludarabine in the Huh-7 cell lines. The expression analysis of CDK2 and CCNA2, and the subsequent cell cycle analysis, threw light on the molecular mechanisms underlying the effects of Imatinib, Fludarabine, and Etravirine on Huh-7 cells.

Further, the untreated Huh-7 cells served as a baseline with a relative Log Fold Change (Log FC) value of 1 for the expression of CDK2 and CCNA2. The treatment with selected drugs based on the IC_50_ led to a significant overall decrease in the expression of both CDK2 and CCNA2. Imatinib at 60.4 µM/ml showed a noteworthy decrease in the Log FC values of CCNA2 and CDK2, indicating a substantial downregulation of these important cell cycle regulators. Similarly, Fludarabine and Etravirine at 120 µM/ml and 36.5 µM/ml, respectively, also resulted in decreased expression of CDK2 and CCNA2. These findings emphasized the need for a comprehensive understanding of the cellular responses induced by Etravirine, particularly in modulating the cell cycle dynamics.

Flow cytometry analysis revealed the effects of Etravirine on distinct stages of the Huh-7 cell cycle. Etravirine at its IC_50_ concentration caused a substantial cell cycle arrest of 13.04% in the Sub G0/G1 phase compared to the untreated group, which exhibited a lower proportion of cellular arrest of 1.38%. In the S phase, Imatinib and Etravirine treatment resulted in a distinguished upsurge in the percentage of apoptosis to 16.21% and 7.17%, respectively, compared to 1.76% in the untreated group suggesting the drugs’ potential to interrupt DNA synthesis. In the G2/M phase associated with mitosis, Imatinib-treated cells exhibited a 41.73% arrest, while Etravirine-treated cells showed a 39.13% arrest. The altered expression levels of target genes and significant perturbation in the finely orchestrated mechanisms governing cell division elucidated the anticancer potential of Imatinib and Etravirine.

Consistent with our findings, Xiao et al. demonstrated the anticancer potential of Imatinib against HCC^19^. Online with our results, Etravirine, a non-nucleoside reverse transcriptase inhibitor primarily indicated for HIV treatment in adults and children, is spotlighted as a possible therapeutic agent against ovarian cancer^20^. Epidemiologic data available indicates a seven-fold rise in the prevalence of HCC in HIV-positive patients with hepatitis compared to HIV-negative controls. Additionally, the risk of HCC is significantly associated with viral infections such as HBV and HCV that result in cirrhosis^21,22^. Thus, Etravirine, identified in the current study, may emerge as a promising candidate as it is proven to influence the various checkpoints of the cell cycle by inhibiting cell cycle regulators, with an additional benefit of established antiviral activity, especially in HCC patients with viral infections. While these findings are encouraging, the transition to clinical applications requires rigorous investigation to ensure Etravitine’s efficacy in a more complex biological system.

## Conclusion

This study captured the significant role played by CCNA2 and CDK2 in the HCC cascade. The evidence derived from this study firmly supports the anticancer potential of Etravirine in HCC based on its influence on cell cycle regulation and apoptosis pathways. Further preclinical and clinical research is warranted to confirm our findings, particularly in patients with viral co- infections.

## Methodology

### Phase 1: Identification of potential targets for HCC and pathway analysis

### Omics-based differential gene expression analysis

Gene Expression Omnibus (GEO) database was exploited to retrieve suitable microarray datasets containing differential gene expression data of normal, cirrhosis, and HCC samples with filtering criteria of FDR p-value < 0.05 and log FC > 1. Based on the fixed criteria, **GSE25097**^23^, **GSE6764**^24^, **GSE98620** (GP14951)^25^ and GSE14323^26^ were selected. The significant overlapping Differentially Expressed Genes (DEGs) between normal vs. cirrhosis and cirrhosis vs. HCC among the four datasets were compiled.

### Protein-protein interactions and gene set enrichment analysis

These overlapping significant genes of HCC were fed in the Search Tool for the Retrieval of Interacting Genes/Proteins (STRING) database to identify the PPI interactions, which were further visualized using Cytoscape with proteins as nodes and interactions as edges. The Molecular Complex Detection (MCODE) in the Cytoscape plugin was used to identify the intersecting clusters from the obtained PPI network. Amongst the cross-talk genes, the genes that displayed the highest node degree were predicted as hub genes. Clue Go, a Cytoscape tool, was used to map the shortlisted proteins with physiological and pathological pathways based on Gene Ontology (GO) and KEGG.

### Validation of hub genes

The significance of these hub genes was validated based on their impact on survival analysis. Patient survival analysis and correlation analysis employ pearson correlation statistics to validate the hub genes by using GEPIA2, a web server for integrating RNA sequencing data from The Cancer Genome Atlas (TCGA) and the Genotype-Tissue Expression (GTEx) project. Subsequently, a target-guided drug library was constructed for the substantial targets associated with poor survival.

### Phase 2: Construction of drug library

A thorough literature search was performed to collate the FDA-approved drugs acting on shortlisted targets. A drug library was created with drugs exhibiting structural and side effect similarities with those of the standard or literature-derived drugs. Herein, the Drug Bank^27^, ChEMBEL^28^, and PubChem databases^29^ were mined to retrieve the drugs with structural similarity based on their Tanimoto index with a cut-off value of 0.5 and 50 percent similarity. Drugs with side effect similarities were captured from LINCS^30^ and SIDER^31,32^ considering the labeled side effects of standard or literature-derived drugs.

## Phase 3: In-silico analysis

### Screening of drug library against targets

The x-ray crystallographic structure of the target proteins was retrieved from the Protein Data Bank (PDB). The drugs from the drug library were considered as the ligands and screened against the shortlisted targets via molecular docking (Schrodinger drug-design suite). Initially, the drug libraries were subjected to a High Throughput Virtual Screening (HTVS) and then docked in Standard Precision (SP) and Extra Precision (XP) mode. The drug-target complexes were shortlisted based on the interactive patterns exhibited by the drugs within the binding pockets of the targets and the binding free energy calculated by MM-GBSA analysis.

### Molecular dynamics

The drugs with high docking scores, better interactions, and binding free energy were further subjected to molecular dynamics via Desmond [Schrödinger Release 2021-2: Desmond Molecular Dynamics System, D. E. Shaw Research, New York, NY, 2021]. A TIP3P solvent model under normal pressure (1 bar) and temperature (300k) ensemble was prepared to develop the molecular dynamics system. The stability of the drug-target complex for 100ns is described in terms of Root- Mean Square Deviation (RMSD), Root-Mean Square Fluctuation (RMSF), and Radius of Gyration (rGyr). The drugs that showed better stability were further investigated by in vitro assays.

## Phase 4: In-vitro analysis

### Cell line and cell culture 

The Huh-7 cell lines were procured from the National Centre for Cell Science (NCCS), Pune, India, and cultured in Dulbecco’s Modified Eagle’s Medium (DMEM) (Cat No: AL111, Himedia) supplemented with 10% Fetal Bovine serum (FBS) and 1% antibiotic-antimycotic solution and incubated at 37°C with an atmosphere of 5% CO2 and 18-20% O2. The cells were maintained through sub-culturing, and those in the exponential growth phase were used for the cytotoxicity assay.

### Cytotoxicity assay

Cell suspension of 100μl was seeded onto 96-well plates and various concentrations of repurposable drugs, such as 12.5, 25, 50, 100, and 200 μM/ml, were added, and the cells were incubated for 24 hours at 37°C with 5% CO2. Then, 3-[4,5-dimethylthiazol-2-yl]-2,5 diphenyl tetrazolium bromide (MTT) reagents were added to the whole volume at a final concentration of 0.5mg/mL and re-incubated for 3hours. The absorbance was measured by using a spectrophotometer reader at a wavelength of 570 nm^33^. The drugs that showed substantial cytotoxicity were selected for further studies.

### CDK2 inhibitory assay

Enzyme-Linked Immunosorbent Assays (ELISA) assay is used to identify the activity of the protein in the presence of shortlisted repurposable drugs. After treatment with IC50 concentration of drugs, the cells were harvested and centrifuged at 2000 rpm. Subsequently, 100μl of biotin-labeled antibody working solution, 100μl of streptavidin-biotin complex (SABC) solution, and 90μl of TMB substrate were added to the supernatant of lysed cells. Read the absorbance at 450nm in a microplate reader^34^.

### Gene expression analysis

The effect of repurposable drugs on the expression of CCNA2 and CDK2 (the shortlisted target from this study) was measured using Reverse transcription polymerase chain reaction (rt-PCR). Cells were cultured in a 6-well plate at a density of 0.5 x 106 cells/ml and were incubated for 24hrs. Then, the cells were exposed to repurposable drugs at their IC50 concentrations, and total RNA was isolated (2μg), then reverse-transcribed to complementary DNA (cDNA) with the IScript cDNA synthesis kit (Cat No.1708890, Bio-Rad, Canada) as per the manufacturer's instruction. The rt-PCR (Quanta Studio3 system, Cat No.272322821, Thermo Fisher Scientific) was carried out in triplicate by using the reaction mixtures comprising 1µL cDNA, 12.5µL SYBR Green Master Mix (2X) (Sensifast SYBR HiRoxkit, Bioline, USA),1µL of each forward and reverse primer (10µM), and 9.5µL of nuclease-free water. Primers used for the study were self-designed using the Primer blast Program developed by NCBI and procured from Eurofins Genomics, Bangalore.

### Cell Cycle Analysis

Cells were treated with repurposable drugs at IC50 concentrations and incubated for 24 hours. Cells were collected by trypsinization and fixed with 70% ethanol. Subsequently the cell pellets were rinsed and resuspended with PBS containing 50μg/ml of propidium iodide in the presence of 100 μg/ml RNase. The distribution of cells in different phases of the cell cycle was determined using a Flow Cytometer (BD FACS Calibur, BD Biosciences, USA)^35^.

## Data Availability

The datasets utilized in our study are publicly accessible through the National Center for Biotechnology Information (NCBI) GEO dataset repository (https://www.ncbi.nlm.nih.gov/geo/query/acc.cgi ). The specific accession numbers are GSE25097, GSE6764, GSE98620 (GP14951), and GSE14323.
